# Complex Linear Response Functions for a Multiconfigurational Self-Consistent Field Wave Function in a High Performance Computing Environment

**DOI:** 10.1021/acs.jctc.3c00317

**Published:** 2023-08-19

**Authors:** Mikael Scott, Mickael G. Delcey

**Affiliations:** † Division of Theoretical Chemistry and Biology, School of Engineering Sciences in Chemistry, Biotechnology and Health, KTH Royal Institute of Technology, SE-106 91 Stockholm, Sweden; ‡ Division of Theoretical Chemistry, Department of Chemistry, Lund University, SE-221 00 Lund, Sweden

## Abstract

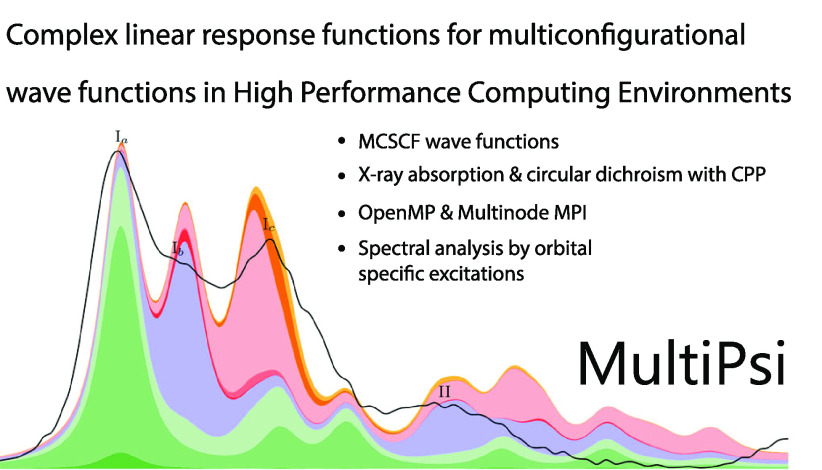

We present novel developments for the highly efficient evaluation of complex linear response functions of a multiconfigurational self-consistent field (MCSCF) wave function as implemented in MultiPsi. Specifically, expressions for the direct evaluation of linear response properties at given frequencies using the complex polarization propagator (CPP) approach have been implemented, within both the Tamm-Dancoff approximation (TDA) and the random phase approximation (RPA). Purely real algebra with symmetric and antisymmetric trial vectors in a shared subspace is used wherein the linear response equations are solved. Two bottlenecks of large scale MC-CPP calculations, namely, the memory footprint and computational time, are addressed. The former is addressed by limiting the size of the subspace of trial vectors by using singular value decomposition (SVD) on either orbital or CI subspaces. The latter is addressed using an efficient parallel implementation as well as the strategy of dynamically adding linear response equations at near-convergence to neighboring roots. Furthermore, a novel methodology for decomposing MC-CPP spectra in terms of intuitive orbital excitations in an approximate fashion is presented. The performance of the code is illustrated with several numerical examples, including the X-ray spectrum of a molecule with nearly one hundred atoms. Additionally, for X-ray spectroscopy, the effect of including or excluding the core orbital in the active space on small covalent metal complexes is discussed.

## 1 Introduction

Multiconfigurational (MC) methods are popular choices to compute excited state properties and spectroscopies, in particular the complete active space self-consistent field (CASSCF),^1^ a specific case of multiconfigurational self-consistent field method (MCSCF) and ensuing correlation treatments, usually using second order perturbation theory, CASPT2 or NEVPT2.^2,3^ These methods are often used as benchmark standards for excited state energies,^4^ are among the best suited methods to describe nonadiabatic dynamics,^5^ and have recently been applied to X-ray spectroscopy,^6^ specifically computing spectra of transition metal complexes to high accuracy.^7^ A key reason for this high accuracy is that, assuming a correct choice of the active space, all orders of excitations (including double and higher excitations) are treated on an equal footing. Those higher-order excitations can contribute significantly to many excited states^4^ and are in particular crucial to describe conical intersections.^8^

Since MCSCF can be thought of as a combination of configuration interaction (CI) and orbital relaxation, there are many ways to compute excited states in a MCSCF wave function depending on how the CI and orbital parts contribute to the excitation of any given state. The most popular method due to its simplicity and relatively good performance is state-averaging. State-averaged MCSCF can be thought of as computing the excited states using only the CI but with a single set of orbitals optimized to be the best compromise between all states. It performs best for a small number of states of similar nature (i.e., which require similar orbitals).

An alternative is to use linear response theory to derive not only the CI but also the orbital response to the field.^9^ In this theory, which can be dubbed time-dependent MCSCF or multiconfigurational random-phase approximation (MC-RPA) by similarity with TD-HF/RPA, excited states are formed as linear combinations of an orbital response (single excitation) and a CI response (up to n-excitations). This removes the spurious dependence of the excitation energies on the number of states, reduces the dependence on the active space, and has been found to give on average more accurate results in a recent benchmark of excitation energies and intensities of organic molecules.^10^

Response theory is a widely applied method to obtain molecular properties by analyzing field-induced changes in expectation values.^11^ Briefly, a known (typically the ground state) reference state is subjected to an external field, and the order of the energy-field derivative determines what property is described by that response function. At first order, one finds the linear polarizability response function, which describes linear absorption and dispersion phenomena as well as circular dichroism. Different response equations may be derived from the linear polarizability response function to obtain either state-specific or transition properties with the most common being the so-called eigenvalue equation for which excited state energies and transition amplitudes are solved, yielding oscillator and rotatory strengths.

Since the eigenvalue approach solves for states, it quickly becomes computationally intractable to solve for energetically high lying excited states, i.e., in the X-ray region or for molecular systems possessing a high density of states. Several techniques have been developed to address this. A common one is the core–valence separation (CVS) approximation which decouples the valence continuum from core-excited states introducing an error to the core excitation energies, which can be corrected for in a postprocessing step.^12,13^ Another method is to use an algorithm for finding interior eigenvalues that lie close to a target excitation energy, for example the harmonic Davidson algorithm, solving a shifted-and-inverted generalized eigenvalue problem which has been introduced at the CASSCF level of theory.^14^ Alternatively one can perform an explicit time propagation of the Dirac–Kohn–Sham density matrix, as done in real-time time-dependent density functional theory (RT-TDDFT), to resolve energetically high lying states.^15^

Here, we focus on another alternative, the so-called complex polarization propagator approach (CPP) where one solves at specific frequencies to calculate the linear polarizability directly.^16−19^ This does not yield state-specific properties, and hence, information about individual excited states is lost. However, the chief benefit of the CPP approach is not requiring solving many roots to compute properties in the higher-energy region. Unlike the CVS approximation, this does not introduce an error, and it tends to have better convergence properties compared to the shifted Davidson method. Additionally, it also removes the need to individually resolve every excited state, which can be beneficial when the density of states becomes high. Hence, one may resolve X-ray absorption and CD spectra using a computational effort similar to that for the lowest energetic roots of a UV/vis absorption or CD spectrum.

Linear response has been applied to Hartree–Fock and density functional theory,^20−22^ and even multiconfigurational wave functions can be used, e.g. CASSCF.^9,23^ For CASSCF, a CPP implementation also exists.^24^ However, this implementation does not exploit separation of gerade and ungerade trial vectors in the shared subspace which has been shown to yield faster convergence.^25^ Additionally, computer power has improved in an exponential manner for the past decades, and as a result, there has been an increasing demand for highly efficient codes which fully exploits the parallel structure of modern high performance computing (HPC) environments.^26^

We recently introduced the program MultiPsi, which enables large scale MCSCF and linear response calculations on HPC environments by building on the foundations of VeloxChem.^27^ The work presented here further extends the capabilities of MultiPsi, specifically by utilizing the CPP scheme to enable highly efficient MC-CPP calculations in the X-ray region. On top of the parallelization of the algorithm, we also suggest novel approaches to solve two known bottlenecks for CASSCF linear response calculations, namely, the memory- and computational-time/effort bottlenecks. MultiPsi tackles the former by making use of a function to collapse the trial vector subspace to refine and limit its size and thereby reduce the memory footprint. In addition, we investigate the idea of adding linear response equations in a dynamic fashion to reduce computational time. Finally, while we lose state resolution, we propose an intuitive way to analyze the CPP spectrum in terms of molecular orbital contributions. All of these developments are tested here on a range of cases, including X-ray calculations. Furthermore, the importance of including core orbitals in the active space on the accuracy of an X-ray spectrum is demonstrated by using this visualization method.

## 2 Theory and Methodology

In this section, we give an overview of the general theory of the CPP applied to MCSCF wave functions, including some of the novel developments in MultiPsi.

### 2.1 Linear Response Functions

The linear (polarizability) response function, also referred to as the standard linear response function, is written as^11^
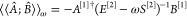
1where *E*^[2]^ and *S*^[2]^ are the electronic Hessian and metric matrices, respectively, *A*^[1]^ and *B*^[1]^ are the property-gradients corresponding to the perturbation operators *Â* and *B̂* acting on the reference wave function |Ψ⟩. When evaluated with the electric dipole as both perturbation operators, eq 1 computes the linear polarizability tensor α_*αβ*_ at external-field frequency ω, where the indices α and β denote spatial dimensions of the *Â* and *B̂* perturbation operators, respectively. In principle, all elements of eq 1 are known when the MCSCF step has converged. The crucial difficulty in its evaluation lies in finding the inverse (*E*^[2]^ – *ωS*^[2]^) ^–1^, which for typical calculations can be on the order of 10^8^–10^14^ elements. Another character of eq 1 is the divergence at excitation frequencies, i.e., when ω = ω_*n*0_, prohibiting evaluation in resonant regions near singularities or “poles”. In the eigenvalue approach, these states are solved for explicitly by the eigenvalue equation
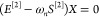
2which is typically solved using a Davidson algorithm to obtain the energetically lowest state energies ω_*n*_, which along with the eigenvector *X* can be used to calculate transition properties, i.e. oscillator and rotatory strengths by proper projection onto the corresponding property-gradients. This has the benefit of revealing the exact-contribution to the linear polarizability or the mixed linear polarizability of a particular excited state but can require a large number of roots to resolve X-ray properties absent the use of CVS approximation or alternative formulations.

As an alternative, the linear polarizability response function has been extended into the complex plane by introducing an imaginary constant *iγ*, called the damping factor, thus yielding the damped (complex) linear response function^28,29^
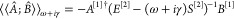
3commonly referred to as the CPP method. Here, γ ensures that singularities are avoided; thus, dynamic or resonant regions can be evaluated. The damping factor γ is inversely proportional to the lifetime of the excited states (assuming identicality for all excited states). The damped linear response function yields the same spectrum as one would obtain from the eigenvalue approach, had all excited states been solved for and a Lorentzian broadening been applied.

From eq 3, one obtains the damped linear response equation
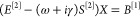
4The solution of eq 4 when contracted with the property-gradient yields the complex linear polarizability tensor
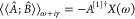
5As mentioned, the main benefit of the CPP method is its direct evaluation at a given frequency or set of frequencies. Additionally, the calculation time does not explicitly depend on the number of underlying states contributing to the spectral region but instead depends only directly on the required resolution.

### 2.2 MCSCF Wave Function Parametrization

The MCSCF reference wave function is written as
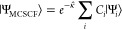
6where κ̂ is the orbital rotation operator, and *C*_*i*_ is the determinant coefficient to the Slater determinant |Ψ_*i*_⟩.^30^ The MCSCF optimization requires both orbital coefficients and CI coefficients. The general expressions for the Hessian and metric matrix elements can be found in ref (9), and our specific implementation MultiPsi is discussed in ref (27). Eq 6 denotes the general MCSCF wave function which incorporates a wide range of active space schemes, e.g., restricted and generalized active space (RAS/GAS) of which the standard complete activate space (CAS) is a special case. In non-CAS cases, the response should in principle include orbital components within the active space to make up for the incompleteness of the excitation space, but here, we neglect these for simplicity.

The Hessian and metric matrices in eq 3-eq 5 have a 2 × 2 block structure corresponding to an excitation and de-excitation part and are in the random-phase approximation (RPA) denoted as^9^
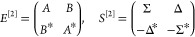
7Commonly the Tamm-Dancoff approximation (TDA) is employed where the B and Δ blocks are neglected which effectively decouples the excitation and de-excitation blocks and thereby significantly simplifying eq 4. The TDA has been observed to generally produce accurate excitation energies but incorrect properties (e.g., oscillator strengths) and fails to adequately reproduce adiabatic coupling elements.^31^

### 2.3 The Damped Linear Response Equation in a Reduced Subspace

One may carry out inversion of the eq 4 left-hand sides to obtain exact solutions directly. However, this quickly becomes computationally intractable for molecular systems consisting of a large number of electrons. Instead one can perform the inversion in a reduced subspace by projection of a basis of trial vectors. Here, one can exploit the fact that the effect of *E*^[2]^ and *S*^[2]^ projected onto a trial vector retains the necessary information to iterate toward an approximate solution.^9^ Specifically, *E*^[2]^ and *S*^[2]^ projected onto a trial vector subspace **b**^*n*^ form so-called σ and τ vector subspaces
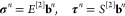
8where **b**^*n*^ is the subspace of trial vectors of size *n*
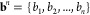
9Because the CI and orbital part of the response equations have different convergence rates, it is customary to separate trial vectors into pure orbital or pure CI vectors^32^
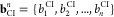
10
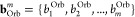
11The total subspace of the trial vector is thus composed of both components
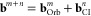
12This projection of the trial vector subspace onto the Hessian and metric matrices needs only be carried out once per iteration where only the new trial vectors are computed.

Using these σ and τ vectors, eq 4 can be expressed in the reduced subspace as
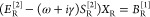
13where the elements of the reduced Hessian *E*_R_^[2]^ and metric *S*_R_^[2]^ matrices are constructed from vector–vector contractions as
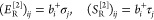
14respectively. Likewise, the property-gradient in the reduced subspace is formed by contraction as
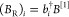
15The solution in full space within a given subspace of trial vectors is then obtained by expansion via back-projection
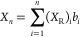
16

From this solution, the residual is calculated by taking the difference with respect to the right-hand side of eq 4
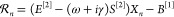
17

If the norm of the residual  is larger than a threshold value, a new trial vector is computed by preconditioning
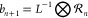
18where *L* is a preconditioner constructed from a diagonal approximation of the electronic Hessian *E*_0_^[2]^. Note that we opt to use the *n*+1 notation here. For the initial guess *b*_1_, the property-gradient is used in place of the residual, *b*_1_ = *L*^–1^ ⊗ *B*^[1]^. As many equations can be solved in each iteration potentially yielding several new trial vectors, the subspace is ensured orthonormal by Gram-Schmidt orthogonalization (*b*_*n*+1_ ⊥ **b**^*n*^) where a linear dependence parameter of 10^–10^ is commonly used. One may also start with a very large threshold and tighten it as needed to remove trial vectors which had a large initial overlap. While this increases the number of iterations, it typically results in a reduced computational cost.

#### 2.3.1 Real Algebra

As noted above, eq 4 yields a complex solution vector. This can be cumbersome dealing with and at no extra computational cost, and one can avoid complex algebra by rewriting eq 4 in terms of two coupled real linear equations as
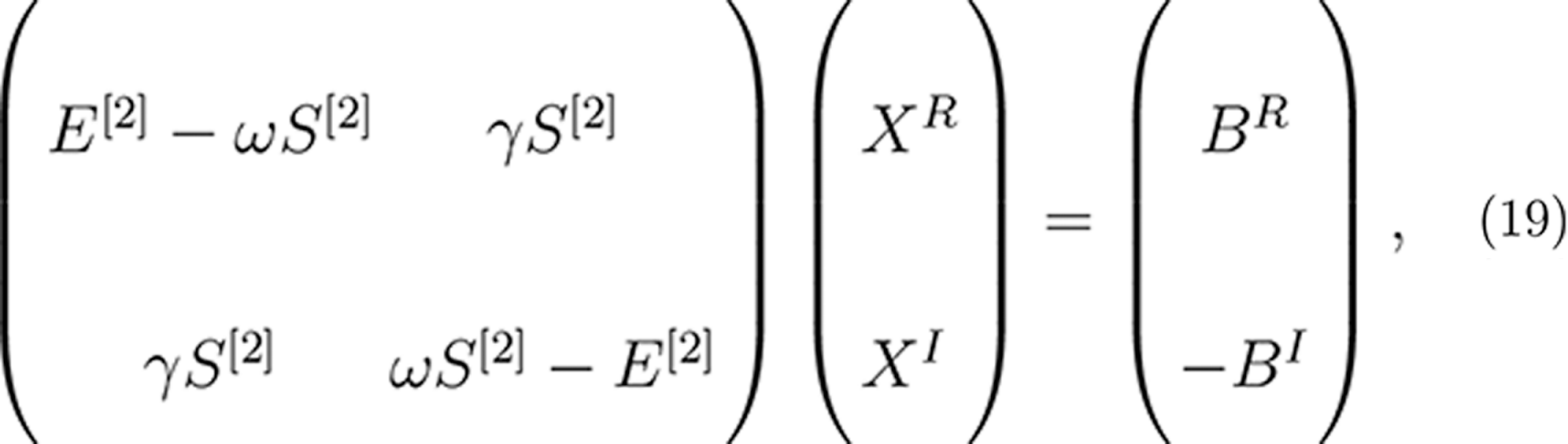
19where *B*^*R*^ and *B*^*I*^ are the real and imaginary components of the property-gradient, and *X*^*R*^ and *X*^*I*^ are the real and imaginary solution vectors.^25^ After eq 19 is solved, yielding the eigenvectors *X*^*R*^ and *X*^*I*^ in the reduced subspace, the full space response vectors are calculated by projection onto the entire subspace (eq 16) as in the complex case. The residual now consisting of two real vectors can then be calculated in an analogous fashion
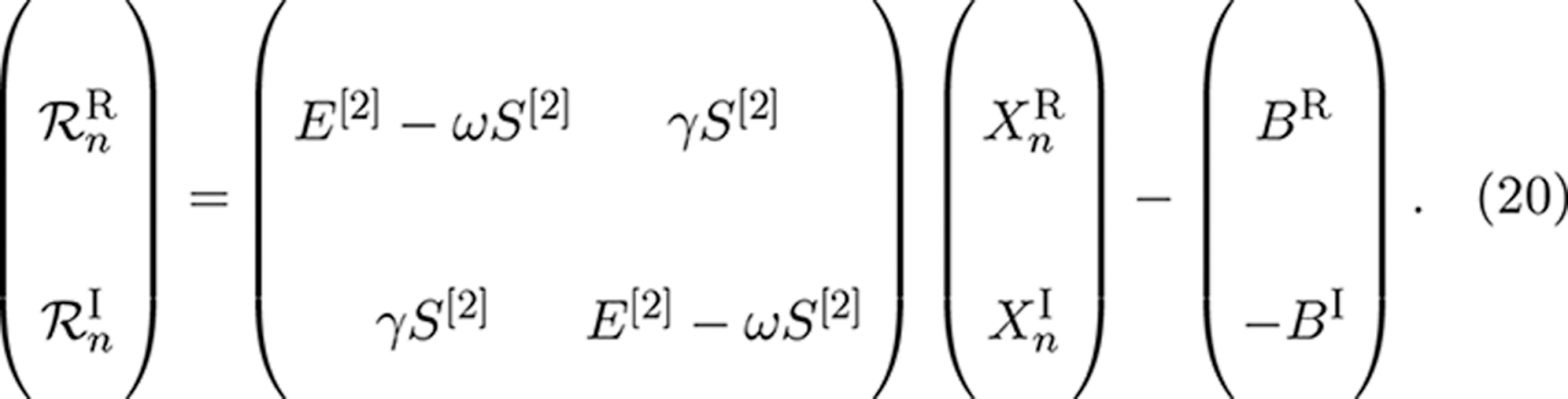
20The total norm of both components  is then taken as the criteria for convergence. If this norm is above a threshold value, two new trial vectors *b*_*n*+1_^R^ and *b*_*n*+1_^I^ are calculated as before by applying a preconditioner
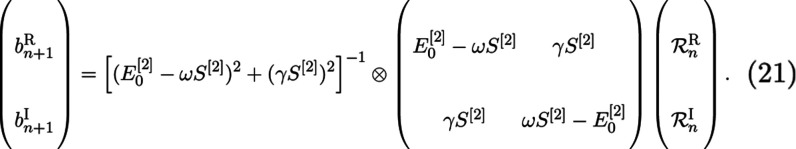
21Both *b*_*n*+1_^R^ and *b*_*n*+1_^I^ are purely real vectors, and the same condition to add them to the subspace of trial vectors as noted for the complex case applies here, namely *b*_*n*+1_ ⊥ **b**^*n*^.

#### 2.3.2 Paired Symmetric and Antisymmetric Trial Vector Approach

For RPA response equations, it has been observed that a faster convergence is obtained when the trial vectors respect the pair structure of the equations, which can be conveniently imposed by decomposing the trial vector space into a symmetric *b*_*g*_ (gerade) and antisymmetric *b*_*u*_ (ungerade) part.^25^ The usefulness of this paired vector approach has further been demonstrated for linear response with MCSCF wave functions^33^ but never been applied to MC-CPP.

The gerade and ungerade trial vectors are defined as
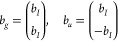
22with *b*_*I*_ being a general CI and/or κ vector.

Projection of a gerade/ungerade trial vector onto the Hessian matrix *E*^[2]^ preserves the given symmetry (gerade/ungerade), whereas projection onto the metric matrix *S*^[2]^ reverses the gerade/ungerade symmetry.

With this partition of the subspace into gerade and ungerade trial vectors, the reduced subspace equation is in the form of four coupled linear equations, which in matrix notation is
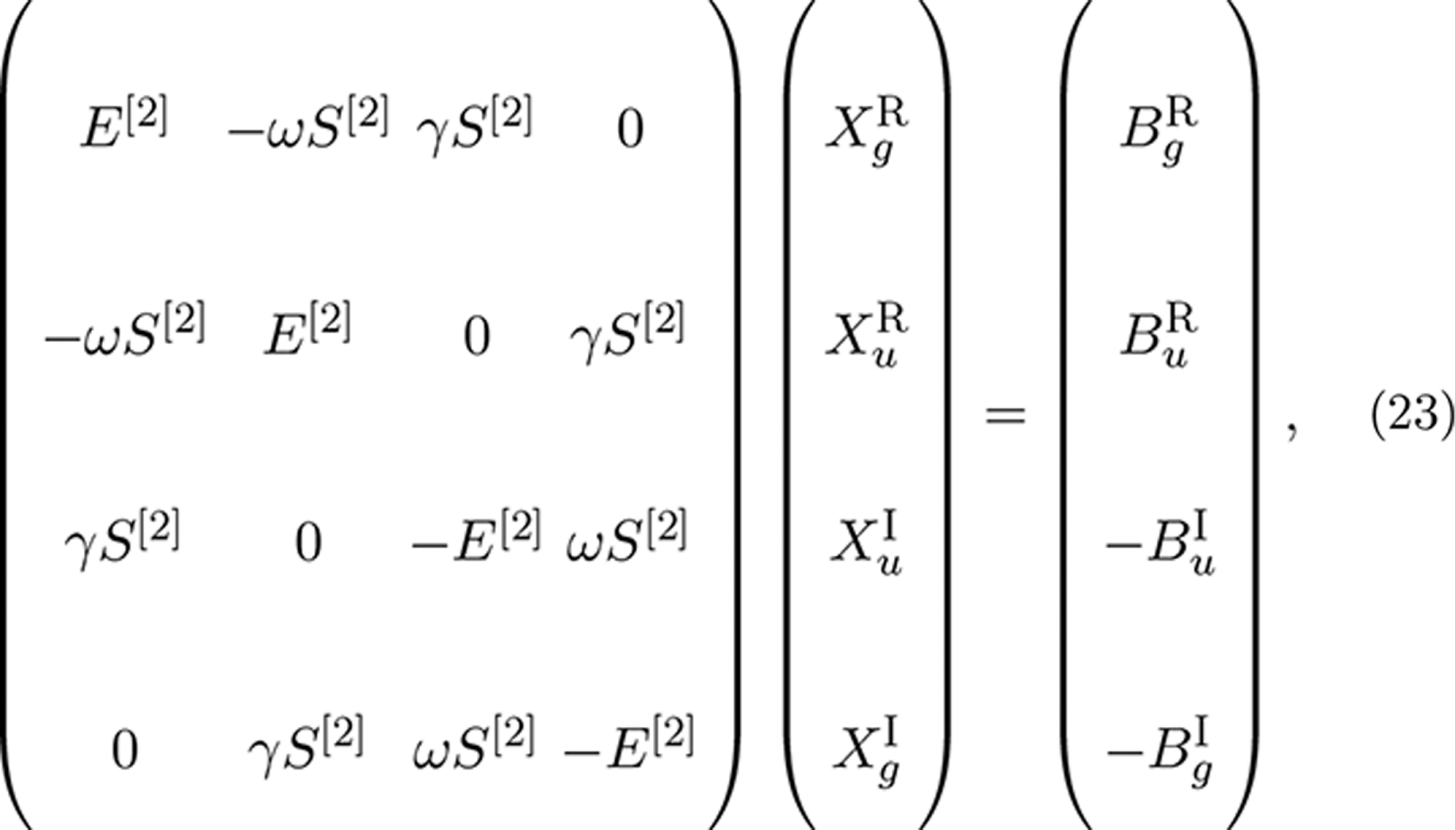
23where *X*_*g*_^R^, *X*_*u*_^R^, *X*_*u*_^I^, and *X*_*g*_^I^ are the gerade/ungerade and real/imaginary solution vectors. In a manner identical to that previously, one obtains these response vectors by contraction with the entire subspace. After these response vectors are obtained, the four-coupled solutions are used to calculate four residuals
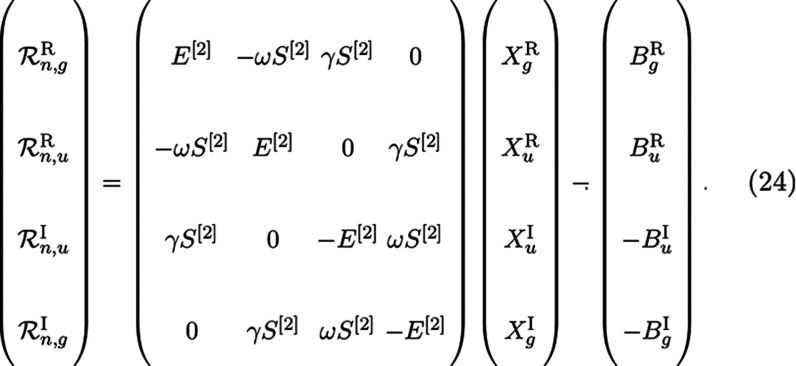
24

Each of these four residuals are then used to construct four new trial vectors as before with a preconditioner which takes the form
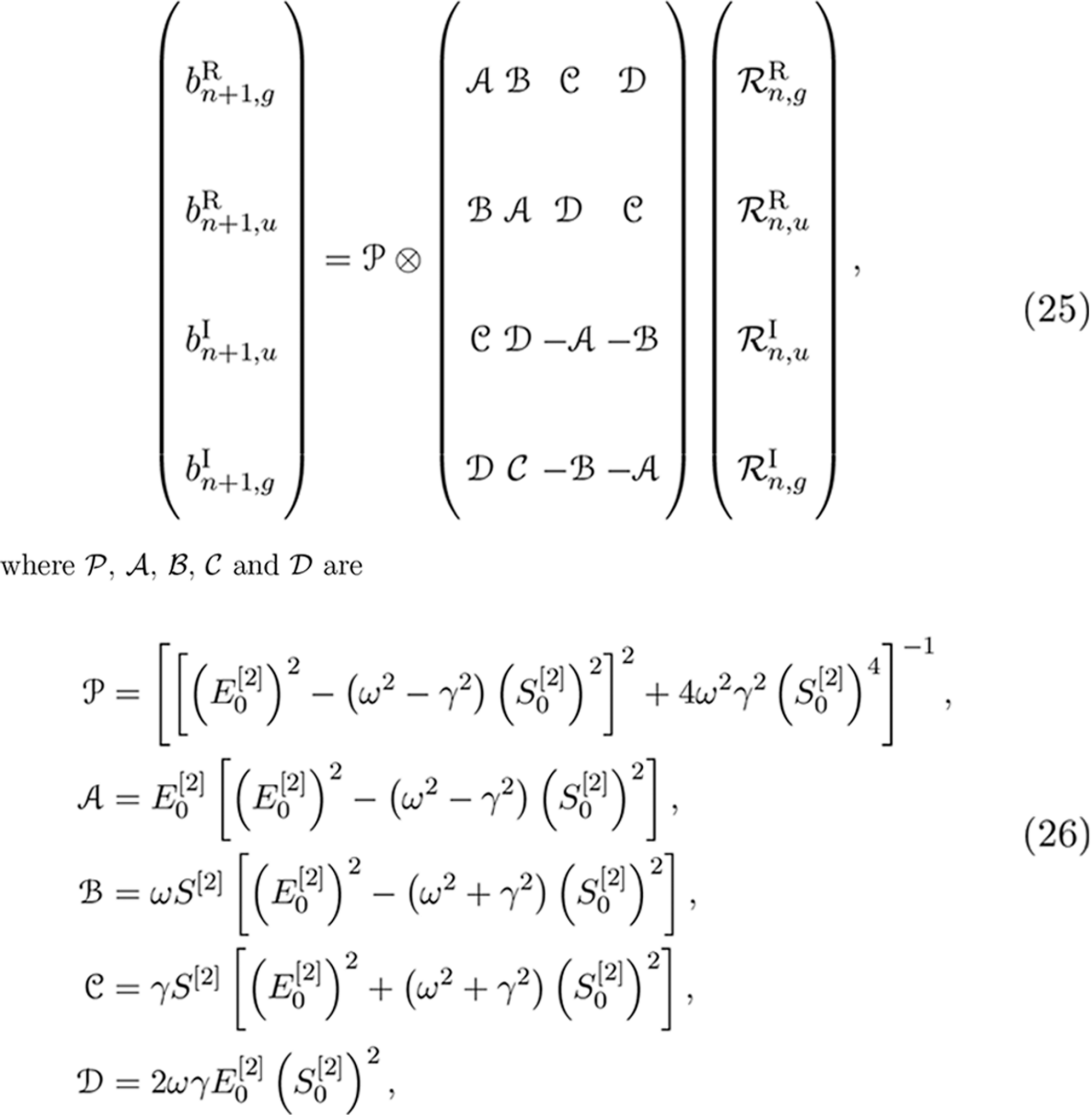
25respectively.^25^ Note that these preconditioner terms are similar to the ones reported in ref (25), but there it was assumed that  = **1** which led to minor simplifications.

Despite being diagonal, these terms can be a strain on memory since the number of them is proportional to the number of frequencies; hence, they are assembled on-the-fly when needed.

In practice, only one common trial vector subspace is kept for which both gerade and ungerade σ vectors are computed at little to no extra cost, therefore obtaining faster saturation of the subspace compared to a separated scheme.

### 2.4 Properties from the Linear Polarizability Response Function

MultiPsi adopts a general-CPP format, allowing for explicit selection of the perturbation operators, *Â* and *B̂*, in eq 3. If the electric dipole operator is used for both *Â* and *B̂*, one obtains the electric dipole linear polarizability tensor α_*αβ*_
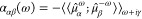
27where the α and β indices denote the Cartesian spatial dimensions. After a response vector *X* has converged in eq 3, contraction with the left property-gradient *A* yields the linear polarizability as
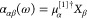
28

Note that α_*αβ*_ here consists of both a real and an imaginary component. From the trace of the imaginary component of the linear electric polarizability tensor α̅, one can calculate the linear absorption cross-section^34^
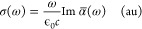
29likewise the trace of the real component Re(α̅) yields the linear dispersion cross-section.

Using the electric dipole operator and the magnetic dipole operator in eq 3 computes the mixed linear polarizability tensor *G*_*αβ*_^′^(ω)
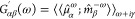
30which is proportional to the electric circular dichroism (ECD) and optical rotatory dispersion (ORD) intensities. For both linear absorption and ECD, the velocity-gauge may be employed using the linear momentum operator for both *Â* and *B̂* in the case of linear absorption and as the *Â* operator in the case of ECD.

Neglecting relativistic effects and remaining within the dipole-approximation, X-ray absorption spectra (XAS) and X-ray circular dichroism spectra (XCD) can be calculated using the MultiPsi package from the above equations.^35^

### 2.5 Analyzing the Excitation Character

When the excited states are solved, it is common to decompose each transition in terms of MO-to-MO specific contributions, thereby giving a more detailed description of the excitation character. This can be done by simply listing the MO-to-MO contributions to the state, or more compactly, by using natural transition orbitals (NTOs), which have been used to describe excitations at CASSCF level.^33,36^ However, the formulation is different for the CPP scheme, and such an analysis has to the best of our understanding not been applied until this implementation in MultiPsi.

For the orbital part, the elements of the property-gradient and corresponding response vector *A*_*n*_^[1]†^ and *X*_*n*_ exist within a known MO-to-MO mapping function ξ
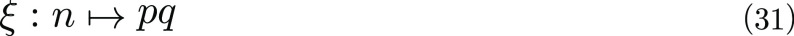
31where *n* is the vector element, and *p* and *q* are molecular orbitals. By exploiting the fact that ξ is known, it is trivially easy to decompose the orbital component of the response derived property into excitation-specific or orbital-specific contributions, e.g. for the linear absorption cross-section
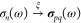
32Here, each element is in principle an explicit molecular orbital-specific contribution from the perturbed MCSCF wave function eq 6, to the linear polarizability eq 3, with the electric dipole operator or the linear momentum operator.

Unfortunately, while the same idea can, in principle, be applied to the CI part, the mapping of the CI expansion to the specific orbital excitation is less straightforward. Thus, while it is perfectly possible for the user to print the CI response vector, for a more intuitive orbital picture, here we suggest using the transition density matrix element
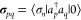
33to approximately map the CI response property to a single excitation from the reference state |0⟩. We note that this definition generalizes eq 32.

This analysis can be performed for each external-field frequency and Cartesian component, but it is significantly easier to interpret the resulting excitation specific spectra if the Cartesian components of the MO specific response are summed to create a partial spectral contribution from this excitation. Thus, instead of a long list, we obtain a full spectrum for each important excitation, which adds up to the total spectrum. In this way, the above equations enable an intuitive molecular orbital picture of the excitation process which is often lacking in propagator methods.

## 3 Numerical Performance

The following section is devoted to looking at the relevant performance behavior of the CPP solver with MCSCF wave functions (MC-CPP) in MultiPsi. For this section, the NSC computational cluster “Tetralith” was used, where each node consists of two Intel Xeon Gold 6130 CPUs each with 16 CPU cores, giving a total of 32 cores (OpenMP) and 96GB memory for 1 node.

**Figure 1:**
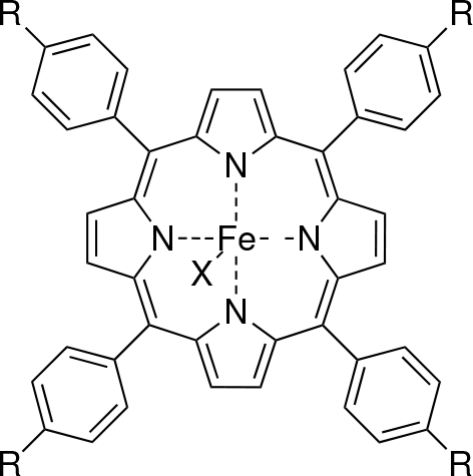
**Porphyrin-A**: X = NO, R = SO_3_^–^; Fe-tetrakis(4-sulfonatophenyl)porphyrin: X = empty, R = SO_3_H.

For evaluating MC-CPP with MultiPsi, the numerical performance was tested using three molecular systems, two large and one small. The small one is the well-known benzene molecule where the active space consists of the ππ* orbitals, corresponding to a CAS(6,6). The second and third are the Fe-tetrakis(4-sulfonatophenyl)porphyrin molecule and one of its derivatives, shown in Figure 1. We denote **porphyrin-A** as the derivative where X = NO and R = SO_3_^–^. For this molecule, the active space is chosen to consist of the iron-ligand σ bonds as well as the Fe-NO π system, corresponding to a CAS(15,11) wave function, whereas for Fe-tetrakis(4-sulfonatophenyl)porphyrin, which lacks the NO moiety, this corresponds to a CAS(7,6) wave function. Since only the numerical performance of the solver was tested, the geometries of benzene and **porphyrin-A** were force-field optimized.

Beyond these two systems, we also demonstrate the excitation character analysis methodology (described in subsection 2.5) in revealing the underlying electronic transition character of the X-ray absorption (XA) spectrum of the K-edge of the O Mn^(III)^O^+^ and Mn^(V)^O_2_^+^, and we further compute the XA spectrum of Fe-tetrakis(4-sulfonatophenyl)porphyrin (Figure 1, X = empty, R = SO_3_H) and compare those results with experimental measurements. For geometrical parameters and detailed information about the active spaces, see the Supporting Information of this article.

For all calculations, a linear dependence threshold of 10^–8^, a convergence threshold of 10^–3^, and a damping factor of 0.124 eV were used if not stated otherwise. Default VeloxChem parameters were used for any SCF step.

### 3.1 Algorithm Structure

The general steps of the MCSCF-CPP algorithm in MultiPsi are

1. Compute intermediates, e.g. half-transformed active integrals for later Hessian *E*^[2]^ contractions (see ref (27)), preconditioner elements *E*_0_^[2]^ and *S*_0_^[2]^, and property-gradients *A*^[1]^, *B*^[1]^.
2. Form an initial guess {*b*_1_} by preconditioning the property-gradient and successively build the subspace of trial vectors by the Gram-Schmidt orthonormalization procedure where vectors are added to within a linear dependence threshold.
3. Main iteration loop over frequencies and spatial dimensions
  (a) Compute and store (new) σ and τ vectors.
  (b) Form and store the reduced subspace matrices *E*_*R*_^[2]^, *ωS*_*R*_^[2]^.
  (c) Solve the reduced subspace equation (*E*_*R*_^[2]^ – *ωS*_*R*_^[2]^)X = *B*_*R*_^[1]^.
  (d) Calculate the residuals .
  (e) If the norm of the residual for any given frequency and dimension is less than the threshold value, calculate linear polarizability. Else, calculate new trial vectors *b*_*n*+1_ and add to the trial vector basis if *b*_*n*+1_ ⊥ **b**^*n*^ to within the linear dependence threshold.
  (f) Collapse CI or orbital subspace if needed.
  (g) For dynamic expansion of the frequency space, add equations if the maximum norm of all residuals is below a given threshold.
  (h) If all equations are converged or a maximum number of iterations are reached or no new trial vectors have been added, exit the main loop.
4. Compute remaining properties, e.g. cross sections or CD intensities.

**Figure 2:**
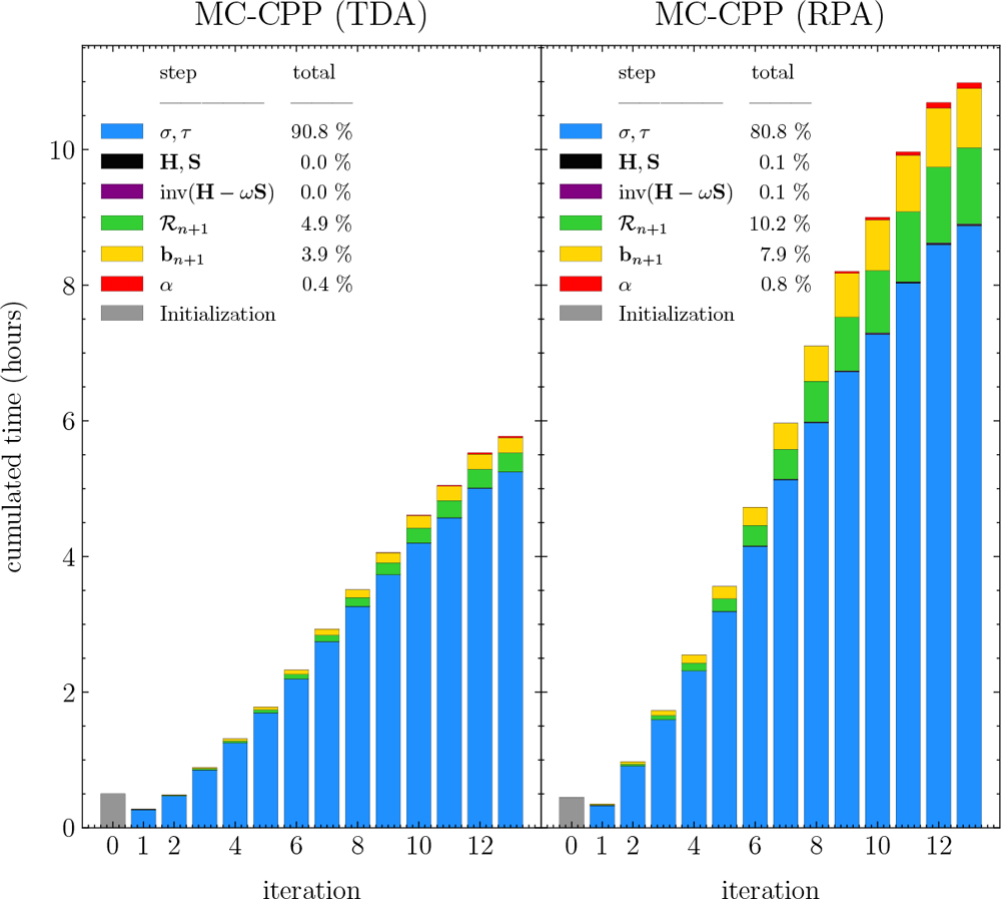
MultiPsi computational time profile showing steps along the algorithm to compute MC-CPP XA spectra of **porphyrin-A** using a CAS(15,11)/def2-SV(P) wave function. The frequency range is 720 to 730 eV at a resolution of 100 frequencies including all spatial dimensions. Initialization incorporates the SCF, MCSCF steps, and the creation of the initial guess.

A typical calculation profile with the time consumed for each step per iteration is shown in Figure 2. Here, MC-CPP is used to compute XA spectra of **porphyrin-A** with a CAS(15,11)/def2-SV(P) wave function between 720 and 730 eV at a resolution of 100 frequencies, with and without the TDA. One obvious observation is that the major time-consuming step is the construction of the σ and τ vectors, which holds true for both the TDA and RPA cases, being roughly 80–90% of the overall computational effort. When tested for even larger cases, this accounted for as much as 95%. All non-σ and non-τ steps take roughly twice to four times as long for RPA compared with TDA, which is in formal agreement with the scaling in terms of the number of equations. However, the cost of computing σ and τ in the RPA is typically less than twice that of TDA due to the fact that computing both the gerade and ungerade σ components can be done at nearly no extra cost, and the number of trial vectors is in practice rarely twice that of TDA due to linear dependency.

### 3.2 Convergence of Linear MCSCF Response Equations

**Figure 3:**
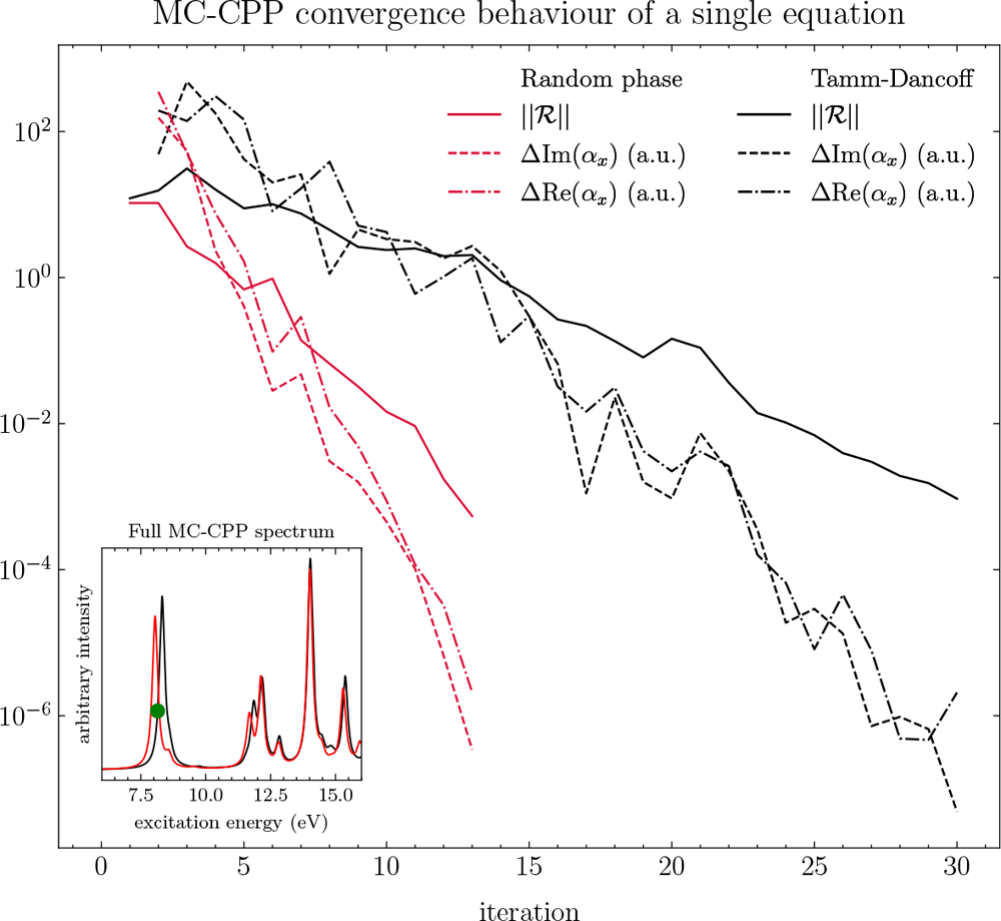
Convergence behavior of a single linear response equation of benzene used with a CAS(6,6)/def2-SVPD wave function, at a frequency of 8.35 eV with and without TDA. The inset figure shows CPP spectra at these same levels of theory with the single equation marked in green.

The convergence behavior of a single MC-CPP response equation was evaluated to ensure smooth convergence of the number of decimals of the linear polarizability with respect to the norm of the residual. Here, benzene and a CAS(6,6)/def2-SVPD wave function were used. A point of high absorbance in the x-dimension, σ_*x*_ = 7.0 (TDA), in the UV/vis region at 8.35 eV (0.3061 au) was selected, and the differential of the linear polarizability between the *n* + 1 and *n*-th iterations, *Δα*_*xx*_^*n*+1^ = α_*xx*_^*n*+1^ – α_*xx*_^*n*^, was computed. The results graphed against iteration are shown in Figure 3.

Here, the MC-TDA case required 30 iterations for the single linear response equation to converge to a residual norm of less than 10^–3^. The change in property *Δα* is roughly proportional to the square of the norm of the residual . This is consistent with previously observed behavior of the linear polarizability tensor obtained via the CPP method, and as a general rule, one obtains 2*n* decimals in the calculated property with a 10^–*n*^ convergence threshold. Hence, MultiPsi MC-CPP uses 10^–3^ as the default convergence threshold parameter to obtain ∼6 decimals in the converged property.

For RPA, a faster convergence was observed, where only 13 iterations are required. This is primarily due to RPA using symmetric and antisymmetric trial vectors where four trial vectors are calculated per equation and iteration, which is twice as many as for TDA. Another caveat is that the high absorbance peak was chosen from the TDA spectrum; hence, the density of states is different in the RPA case. As was the case for TDA, one converges roughly 2*n* decimals with a residual norm of less than 10^–*n*^ for RPA.

Using the same wave function but solving for all spatial dimensions and 100 frequencies between 0.0 and 8.35 eV, roughly 1/3 of the number of iterations are needed, as would be expected when multiple equations were solved in a shared subspace. Slow convergence behavior of linear response equations has been observed in past CAS implementations and was a chief motivation for developing the gerade and ungerade (g/u) subspace approach. While we exclude any comprehensive comparison and discussion regarding previous work, the reader is directed toward the Supporting Information of this article, which demonstrates the benefit of using g/u in contrast to the standard approach.

### 3.3 Collapse of CI and Orbital Subspaces

The computation of new σ and τ vectors is not only the most time-consuming step of the algorithm as demonstrated in Figure 2 but also the main memory bottleneck since they need to be stored in memory and cannot be computed on-the-fly. Because of the strain on memory, a ceiling can be hit leading to a crash. To avoid this, we explore the use of a collapse of subspace function, which refines and limits the size of the subspace.

When the size (number of vectors) of the subspace exceeds a given number, the subspace is reduced to a minimal size that can still represent the current solution(s) with reasonable accuracy. For this, the approximate solutions to eq 4 are placed in matrix , and the SVD is calculated
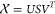
34Here, *U* and *V* are unitary matrices, and *S* is the rectangular diagonal matrix of singular values. Through the screening of small singular values, the trial vector space can be reduced with minimal loss of accuracy. The refined subspace is then obtained as
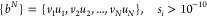
35where 10^–10^ is the default linear dependence threshold. The collapse function can be made even more strict by not including the converged solutions to , since these are no longer solved for, although this typically does not benefit the convergence behavior significantly since neighboring frequencies are built upon a shared vector space.

**Figure 4:**
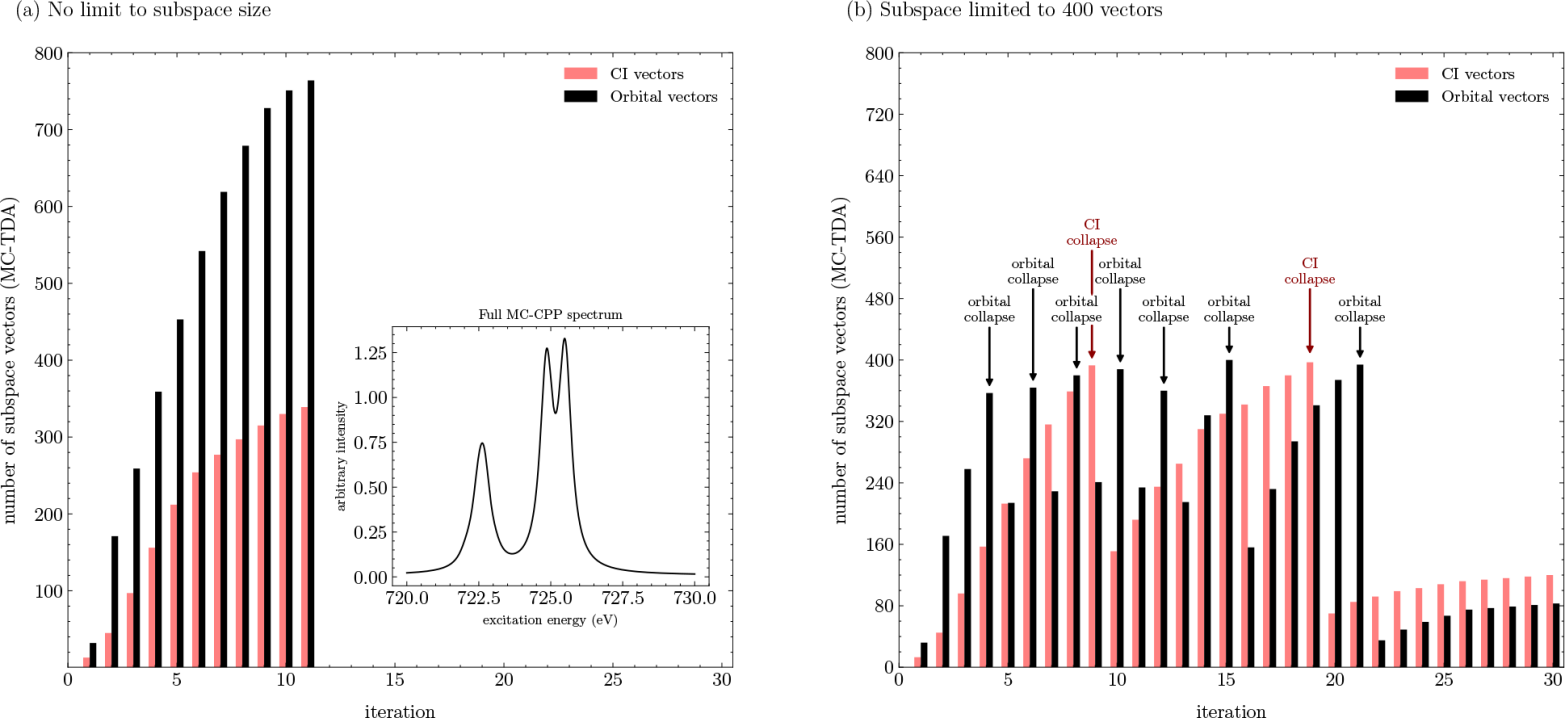
Number of CI and orbital trial vectors in an MC-TDA XA calculation (720 to 730 eV, 100 frequencies, all spatial dimensions) of **porphyrin-A** with a CAS(15,11)/def2-SV(P) wave function when (a) imposing no limit to subspace size and (b) using the collapse subspace function on the CI or orbital subspaces if either exceeds 400 vectors. The inset in (a) is the calculated XA spectrum.

The main downside of using a collapse function is that it artificially constricts the number of trial vectors, thereby negatively impacting the rate of convergence. This behavior is illustrated in Figure 4. Here, the number of orbital and CI trial vectors in the subspace of an MC-CPP calculation of **porphyrin-A** using a CAS(15,11)/def2-SV(P) wave function is shown. Specifically, the wave function consists of 5082 determinants and 200255 orbital parameters. The spectral region is between 720 and 730 eV at a resolution of 100 frequencies including all spatial dimensions, using a damping factor of 0.248 eV, and the computed CPP-spectrum is shown as an inset in Figure 4a. Allowing an unconstrained number of trial vectors requires 11 iterations to converge all equations to a residual norm of less than 10^–3^, Figure 4(a). The orbital subspace reaches a maximum number of 764 vectors, which is roughly 0.38% of the full orbital vector space. The CI subspace reaches a maximum 339 vectors, which is roughly 6.67% of the total CI vector space.

Next, the collapse-subspace function was employed, limiting the number of orbital and CI vectors to a maximum of 400 each (0.2% and 7.8% of the total orbital and CI vector spaces), as shown in Figure 4(b). For this case, 30 iterations are required to converge all equations. The orbital subspace collapses seven times, and with a smaller orbital subspace after each collapse, the CI subspace expands beyond the unconstrained case to more than 400 vectors, collapsing twice. This demonstrates an effective way to limit the subspace size and still obtain converged solutions, effectively dealing with memory-bottleneck issues of MC-CPP calculations, though at an increased computational cost (here roughly 30%).

### 3.4 Dynamically Adding Equations near-Convergence

The main benefit of solving for multiple equations is faster convergence due to the amount of shared trial vectors of each separate equation. Although convergence is reached with fewer iterations compared to solving each equation separately, there is a trade-off with the increased time per iteration. As shown in Figure 2, construction of σ and τ vectors is the most prohibitive step, and solving for fewer equations or more specifically at fewer frequencies enables a more progressive buildup of the trial vector subspace. Here, we explore a dynamic approach where equations are progressively added as equations near convergence since equations of neighboring frequencies will regardless share a large portion of the subspace.

**Figure 5:**
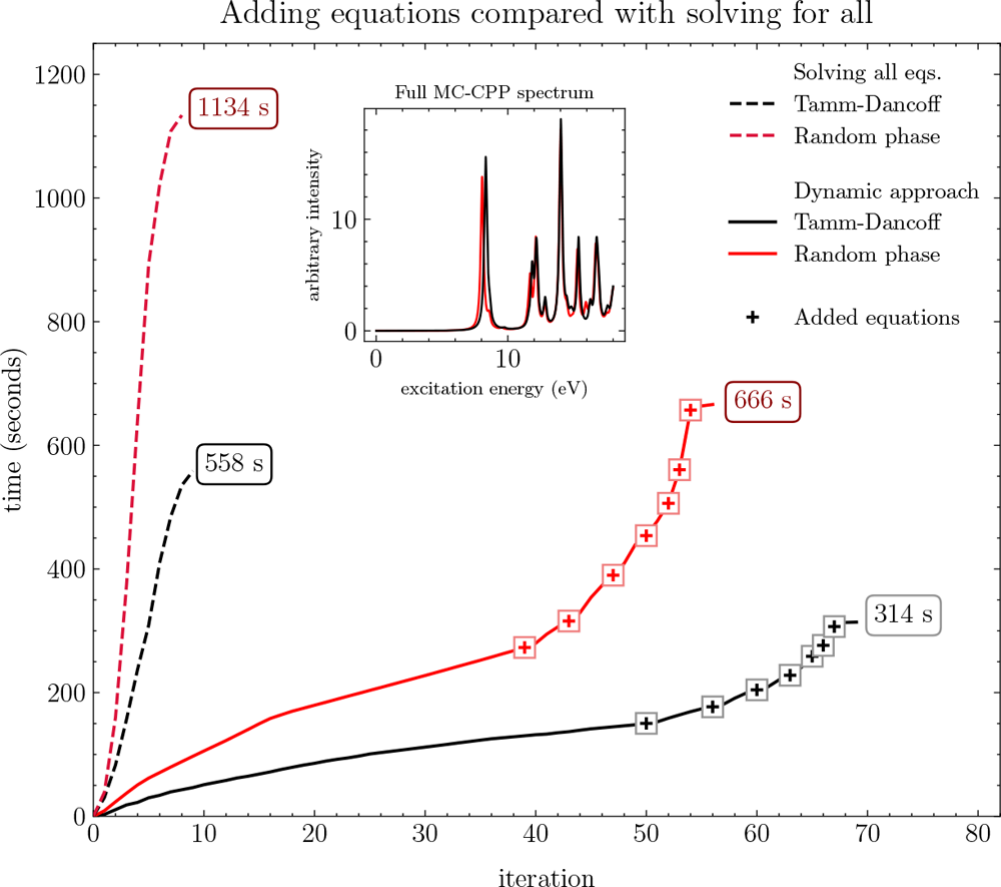
Solving all MC-CPP equations simultaneously (dashed lines) vs dynamically adding equations near-convergence of neighboring roots (solid lines), tested on benzene using a CAS(6,6)/def2-SVPD wave function. The frequency range is 0 to 18 eV at a resolution of 200 frequencies. Markers indicate at which iteration equations were added.

An illustrative demonstration of this approach is shown in Figure 5. Here, MC-CPP spectra of benzene using a CAS(6,6)/def2-SVPD wave function is solved for with and without the TDA. The frequency range is from 0 to 18 eV at a resolution of 200 frequencies including all spatial dimensions. When all equations are solved simultaneously, roughly 10 iterations were required for both RPA and TDA with calculation times (excluding initialization) of 1134 and 558 s, at the TDA and RPA levels, respectively.

If instead four frequencies (evenly spaced over the frequency range) are solved initially with equations at neighboring frequencies added in when the max residual norm is lower than 10^–2^, far more iterations were required to converge all roots. In this approach, TDA and RPA converged in 69 and 56 iterations, respectively. While more iterations were required, the computational times were considerably reduced, finishing in 314 s at the TDA level (43.8% reduction) and 666 s (41.3% reduction) at the RPA level of theory.

Furthermore, the impact of different initial frequency spaces and residual norm conditions was tested for the same CPP calculation. Specifically, three different numbers of starting frequencies, two, four, and eight, are tested (spaced homogeneously) as well as three threshold conditions for the max residual norm, 10°, 10^–1^, and 10^–2^, were used. The reduction in computational time is shown in Table 1. While there is an impact on the various calculation conditions, there is consistency in the reduction of computational effort, which is reduced by roughly 30–40% at both the TDA and the RPA levels of theory. A further benefit of this approach is that the resolution of a computed CPP spectrum can be higher without a major increase in the computational effort. As the subspace becomes sufficiently saturated to converge an entire frequency range, added equations are instantly converged, and hence, only the reduced subspace equation accounts for additional computational effort, which is already negligible (Figure 2).

**Table 1:** Reduction in Computational Effort Using Dynamic Addition of Equations by Two Criteria, Number of Initial Frequencies and Max Residual Norm Threshold, to Add Neighboring Equations^b^

		Time reduction (%)		Iterations	
No. of initial frequencies	Add at	TDA	RPA	TDA	RPA
2	0	30.9	36.1	61	50
2	1	43.1	37.9	76	61
2	2	43.4	41.5	90	67
4	0	33.3	35.1	47	39
4	1	43.7	42.1	58	46
4	2	43.8	41.3	69	56
8	0	34.9	35.5	33	29
8	1	42.7	38.0	40	31
8	2	47.9	41.5	46	37
a200	–	0.0	0.0	9	8

a Full frequency space, i.e. all equations solved simultaneously.

b Tested on benzene using a CAS(6,6)/def2-SVPD wave function for CPP spectra between 0 to 18 eV at a resolution of 200 frequencies.

While these results are promising, it is obviously somewhat system dependent, and molecular systems for which a majority of spectral regions share common subspace vectors would benefit the most.

### 3.5 Parallel Performance

Central to our MC-CPP code is the ability to scale with both number of cores when running on a single node and scaling with the number of nodes in a multinode architecture which is the *de facto* standard in modern high performance computational clusters. The main details are identical to the standard linear response implementation already described in MultiPsi.^27^ The code is fully conventional (i.e., no integral approximation beyond standard screening), but its key feature is the use of the integral-direct and highly parallelized multiple Fock matrices constructor of VeloxChem.^26^ In summary, the main step in the orbital-σ vector computation is the construction of Fock matrices with one-index transformed densities. Two Fock matrices (inactive and active) are needed for each orbital trial vector, and the densities and Fock matrices are distributed across Message Passing Interface (MPI) ranks, while the Open Multi-Processing (OpenMP) multithreading is inherited from that of the VeloxChem Fock matrix constructor. Most of the other needed matrices are computed from the MO integrals with two active indices which are computed at the beginning and stored in the distributed memory. These integrals are also computed using the Fock matrix constructor and distributed densities across MPI ranks.

**Figure 6:**
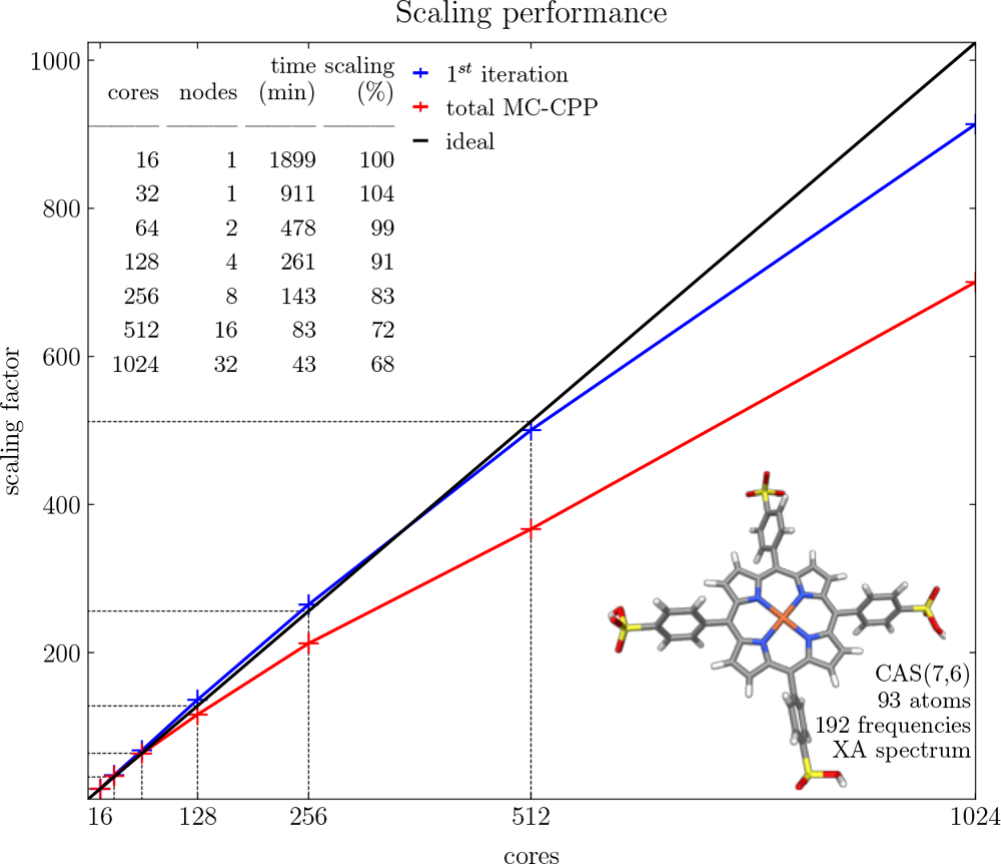
Scaling performance of the MC-CPP algorithm in MultiPsi tested on Fe-tetrakis(4-sulfonatophenyl)porphyrin using a CAS(7,6)/def2-SV(P) wave function solving for an XA spectrum (between 410 and 417 eV at a resolution of 192 frequencies). Sixteen OpenMP threads (half a node) are used as a starting point, and then the number of MPI ranks is increased to reach 32 full nodes (1024 cores).

In addition, the CI and orbital trial vectors are scattered across MPI ranks, which not only helps with memory but also, in effect, provides MPI parallelization of the main steps outside of the σ construction. To test the scaling performance of the MC-CPP solver of MultiPsi, an XA spectrum consisting of 192 frequencies between 410 and 417 eV including all spatial dimensions of the Fe-tetrakis(4-sulfonatophenyl)porphyrin molecule with a CAS(7,6)/def2-SV(P) wave function were solved for using a varying number of OpenMP threads and MPI nodes. The resulting scaling factor with respect to the number of cores is shown in Figure 6. At the lower end using 16 OpenMP threads, the total MC-CPP calculation time was 31 h, 39 min, and 11 s. Using 32 cores (the full node), the calculation time was 15 h, 1 min, and 10 s, which corresponds to 104.2% efficiency toward ideal scaling.

Employing multiple nodes retains this efficiency, achieving 99.3% and 90.8% scaling at two and four nodes, respectively. Using 8, 16, and 32 nodes, corresponding to 256, 512, and 1024 cores, shows a drop in efficiency with the latter achieving 68.4% scaling, resulting in the fastest MC-CPP computational time of 43 min and 23 s. The reason for the lower efficiency can be understood if one considers only the first iteration (blue line in Figure 6). For this iteration, we observe a scaling efficiency between 90% and 105%, i.e., near ideal. The time for this iteration corresponds almost exclusively to the σ construction which is the most optimized part of the code. The other steps, solving of the reduced subspace equations, calculation of residuals, and new trial vectors, are minor in comparison but less well parallelized and start to become significant with an increasing number of nodes. However, since the fraction of time spent in the σ construction increases with system size, larger systems would show even better parallelization efficiency.

### 3.6 Active Space Choice for X-ray Spectroscopy and Excitation Character Analysis: Manganese Oxide Example

One of the strengths of the CPP approach is the ability to quickly resolve molecular response in the high energy regions, in particular, soft and hard X-rays. Additionally, unlike in the state-averaged approach, the linear response formulation does not require the core orbitals to be in the active space, thus, significantly reducing the computational cost. However, it is still possible to include these orbitals, which can be beneficial since these excitations will now be handled by the more accurate CI part instead of the orbital part. Here, we illustrate this in two numerical examples, namely the O K-edge XA spectra of Mn^(III)^O^+^ and Mn^(V)^O_2_^+^. The calculations were done at the CASSCF level first and then at the RASSCF level, including the core orbitals in the RAS1 space allowing a single excitation from this space. While it can be challenging to converge such a RASSCF wave function without the core orbitals rotating out of the active space, we could do it without using constraints as long as we started from already converged CASSCF.

The results were compared to high resolution experimental spectra, and the excitation analysis methodology detailed in subsection 2.5 was used to illuminate the underlying electronic transitions.

Transition metal-oxo species serve as intermediates in water oxidation reactions, as well as a range of biological functions, e.g. in Photosystem II where manganese-oxides are the reactive species.^37^ The Manganese-oxide complexes, Mn^(III)^O^+^ and Mn^(V)^O_2_^+^, have previously been examined using the state-averaged RASPT2 method using the CVS approximation to probe the O K-edge as well as the Mn L_2,3_-edge for their XA spectra.^38^ While that yielded results consistent with experimental measurements, it was computationally expensive since a large number of states needed to converge. As a demonstration of the computational efficiency afforded by the CPP approach, the same spectra are recomputed here. The geometries of both molecules were adopted from ref (38).

**Figure 7:**
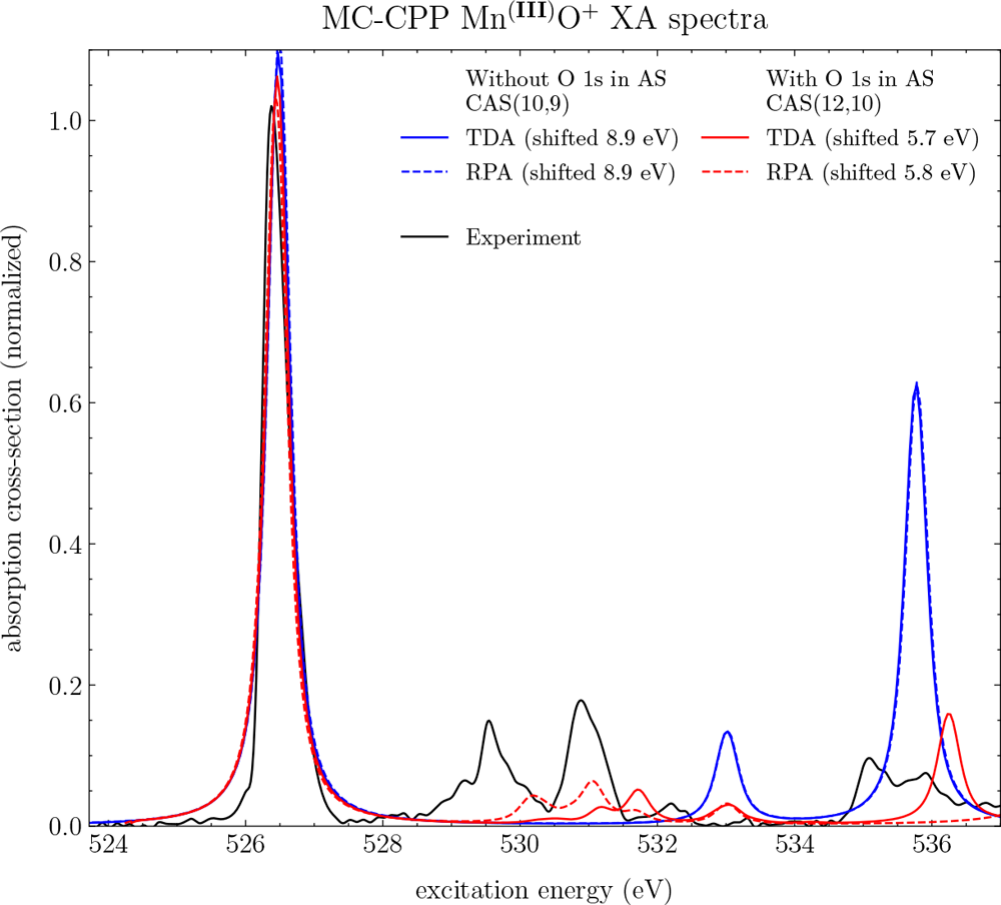
MC-CPP XA spectra of Mn^(III)^O^+^ between 526 and 540 eV at a resolution of 300 frequencies with and without TDA. Using a wave function without the O 1s in the active space, corresponding to a CAS(10,9) (blue), with the O 1s in the active space, corresponding to a RAS(12,10) (red). A damping constant of 0.16 eV was used, and post-CPP, a Gaussian broadening function with a HWHM of 0.1 eV was applied. The experimental line (black) is taken from ref (38).

Shown in Figure 7 are the theoretical XA spectra of Mn^(III)^O^+^ calculated at the MC-TDA and MC-RPA levels of theory using two different MCSCF wave functions with the def2-SV(P) basis set. A 0.16 eV damping factor was used in the CPP calculations, and subsequently, a Gaussian broadening function with a HWHM of 0.1 eV was applied to the converged spectra obtaining a final Voigt profile spectral shape. In one case, the active space consists of a CASSCF with the full valence space without the O 1s orbitals, corresponding to a CAS(10,9). Using this wave function, the shifts required to align with experiment are roughly 9 eV for both TDA and RPA. The major peak in the O K-edge is reproduced, though the remaining peaks at 530 and 531 eV are either heavily blue-shifted or not present.

However, thanks to the general CI code of MultiPsi, the core orbitals can be included in the active space using a RASSCF^39^-like wave function with a single excitation from these core orbitals, which will be called thereafter RAS(12,10). Doing this, a major improvement in the theoretical excitation energy of the O K-edge is observed. Here, a smaller shift is required to align the theoretical spectra with the experimental one, at 5.70 and 5.77 eV for MC-TDA and MC-RPA, respectively. The experimental spectral features at 530 and 531 eV are at these levels of theory reproduced, although lower in intensity as compared to the experiment and slightly blue-shifted.

The impact of including the oxygen core orbital in active space can easily be understood. Unlike state-averaged MCSCF, excitations outside of the active space are treated here at the single-excitation level. This has two implications, first a neglect of relaxation effect, which explains both the larger energetic shift and overall spectral shape. Second, since double or higher order excitations are assumed to be at the origin of the weak satellite peaks, these are neglected without inclusion of the O 1s orbital in the active space. It should be noted that this is a highly covalent metal complex and thus represents an extreme case. For a plurality of other similar metal complexes, excluding the core orbitals from the active space will yield reasonable results, often at significantly reduced cost.

**Figure 8:**
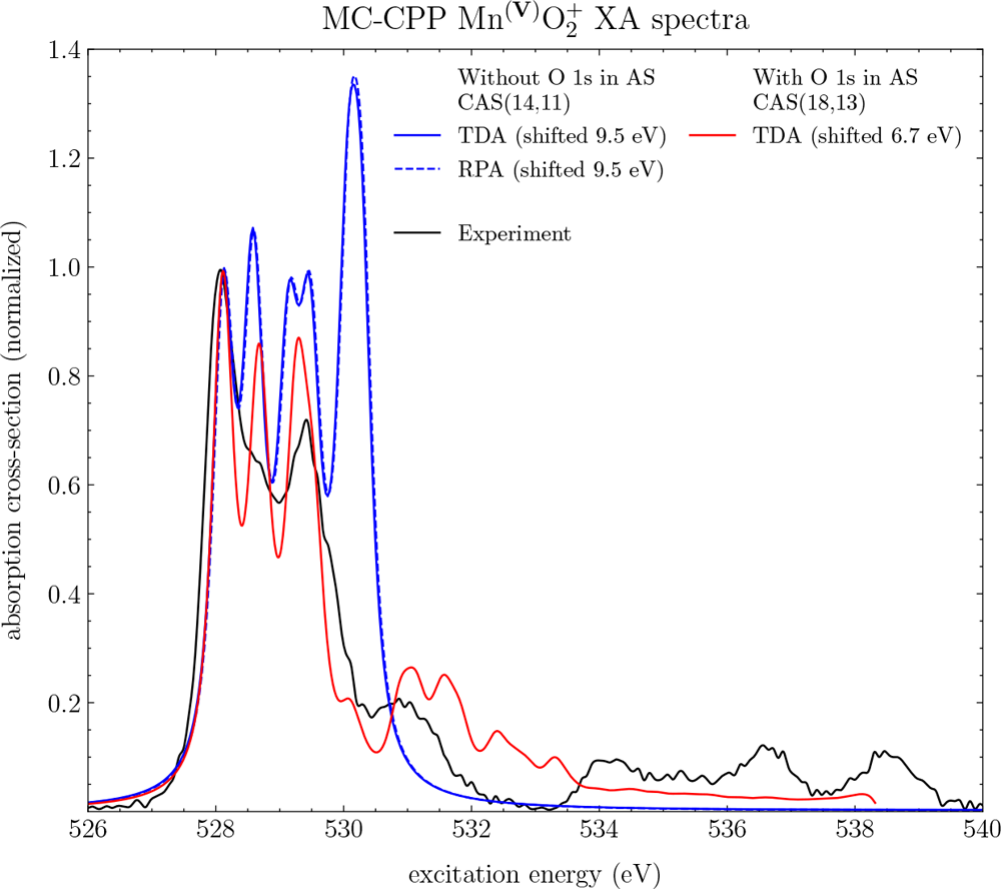
MC-CPP XA spectra of Mn^(V)^O_2_^+^ between 526 and 540 eV at a resolution of 300 frequencies with and without TDA. Using a wave function without the O 1s in the active space, corresponding to a CAS(14,11) (blue), with the O 1s in the active space, corresponding to a RAS(18,13) (red). A damping constant of 0.16 eV was used, and post-CPP, a Gaussian broadening function with a HWHM of 0.1 eV was applied. The experimental line (black) is taken from ref (38).

Turning to Mn^(V)^O_2_^+^, the XA spectra at the MC-TDA and MC-RPA levels of theory are shown in Figure 8, where the same damping factor and Gaussian broadening have been used. Here, the intense part of the experimental spectrum is more complex, constituted of several peaks between 528 and 531 eV. Without inclusion of the oxygen core orbitals in the active space, the agreement is already reasonable at both the TDA and RPA levels of theory (after a shift of 9.5 eV was applied), although the energies and intensities of the higher energy peak in the main band are significantly higher than the experiment. Additionally, as for Mn^(III)^O^+^, several lower intensity features are absent when excluding O 1s from the active space, namely the shoulder at 531 eV as well as the rather weak but broad spectral feature between 534 and 540 eV.

Inclusion of the O 1s orbitals in the active space yields a significant improvement in both the spectral shape of the intense band feature and the shoulder feature at higher energies. A smaller shift of 6.7 eV is required to align with experiment. Furthermore, the broad spectral feature after 534 eV is somewhat reproduced, although at a significantly weaker intensity without its distinguishing features.

**Figure 9:**
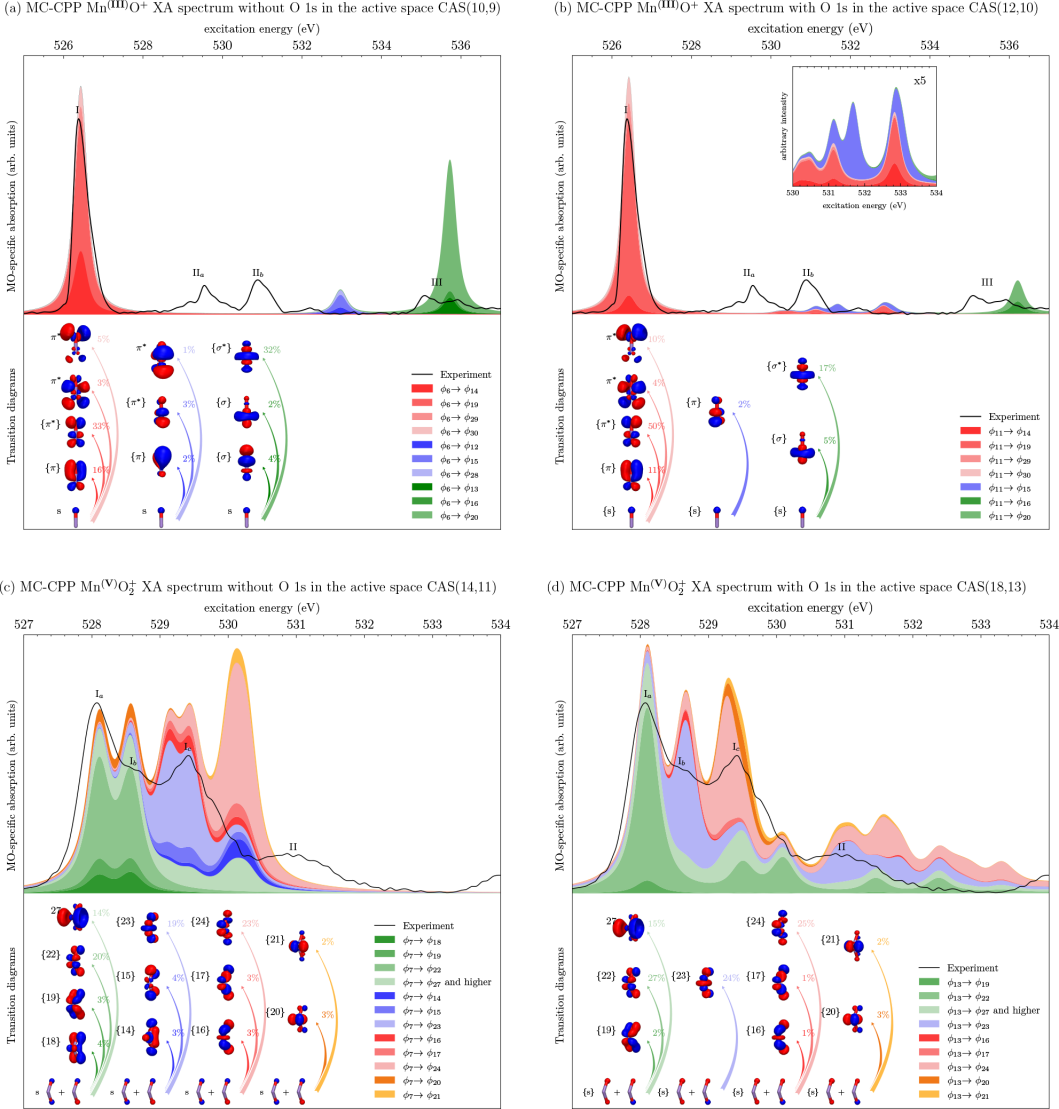
Excitation character decomposition of Mn^(III)^O^+^ RPA (top) and Mn^(V)^O_2_^+^ TDA XA K-edge spectra (bottom) where (a, c) are without inclusion of the O 1s orbital in the active space and (b, d) are with inclusion of the O 1s orbital in the active space.

By utilizing the excitation character analysis methodology outlined in subsection 2.5, the individual MO-specific contributions to the MC-RPA XA spectrum of Mn^(III)^O^+^ can be resolved, as seen in Figure 9a and Figure 9b. The percentages (weights) shown next to the transition diagrams are normalized values, considering only those contributions shown in the figure. At the RPA level of theory, the energetically lowest peak in the XAS spectrum, I, is clearly dominated by *s* → π* excitations with or without inclusion of the O 1s orbital in the active space. It is interesting to note the presence of contributions from the higher lying orbitals of π symmetry formed from the Mn 4d and O 3p orbitals. Those orbitals, while not included in the active space, contribute to the relaxation effect. In a state-averaged calculation, those orbitals may partly enter through orbital optimization but only in an approximate way, which illustrates one advantage of using linear response over state-averaging.

The next spectral features are the two narrowly separated satellite peaks, marked II_*a*_ and II_*b*_, centered around 530 and 531 eV. At the MC-RPA level of theory, these are also *s* → π* transitions but in the perpendicular plane (which are nondegenerate due to different occupations), although they are not resolved as two separate peaks without inclusion of the O 1s orbitals in the active space but rather as a single blue-shifted peak centered around 533 eV. We remind the reader that a caveat to this analysis is that the CI part is truncated at single excitations only, eq 33, which does not give a complete picture of the excitation character especially for these satellite peaks.

Next, we perform the same excitation analysis for the XA spectra of Mn^(V)^O_2_^+^ calculated at the MC-TDA level of theory, as shown in Figure 9(c) and Figure 9(d). The largest feature in the experimental spectrum is the main band centered around 528 to 530 eV itself consisting of two subpeaks, denoted *I*_*a*_ and *I*_*c*_, as well as a smaller spectral peak in between, denoted *I*_*b*_. Without inclusion of the O 1s orbitals in the active space, all three features are characterized by three transitions to the main valence antibonding orbitals with weaker contributions from excitations into singly occupied d orbitals. Each of these three MO-specific absorption bands consist of two peaks, separated by a narrow gap of 0.7–0.2 eV. As evident from the MC-TDA spectrum shown in Figure 8, the shoulder is not reproduced. However, when the O 1s core orbitals are included in the active space, the shape and position of these transitions are altered, from two narrowly separated peaks to a single peak each. This decomposition assigns the higher energy features to convolutions of transitions from these same orbitals, but they likely have significant double excitations character, explaining their absence in the calculation without inclusion of core orbitals in the active space.

### 3.7 Large-Scale Examples: N K-Edge XA Spectrum of Fe-tetrakis(4-sulfonatophenyl)porphyrin

As a further demonstration of the performance of MultiPsi, the N K-edge XA spectrum of Fe-tetrakis(4-sulfonatophenyl)porphyrin was calculated. This molecule serves as an excellent test case for numerical performance owing to its large size, as well as evaluation of the accuracy of an MCSCF method owing to the strongly correlated electronic structure arising from the iron-ligand core. Furthermore, a high quality experimental XA spectrum has previously been published.^40^ The active space for this calculation was chosen to include the iron 3d orbitals as well as the ligand-centered iron–nitrogen σ MO, corresponding to a CAS(7,6)/def2-SV(P) wave function, which gives a response calculation with 189429 orbital and six CI parameters.

**Figure 10:**
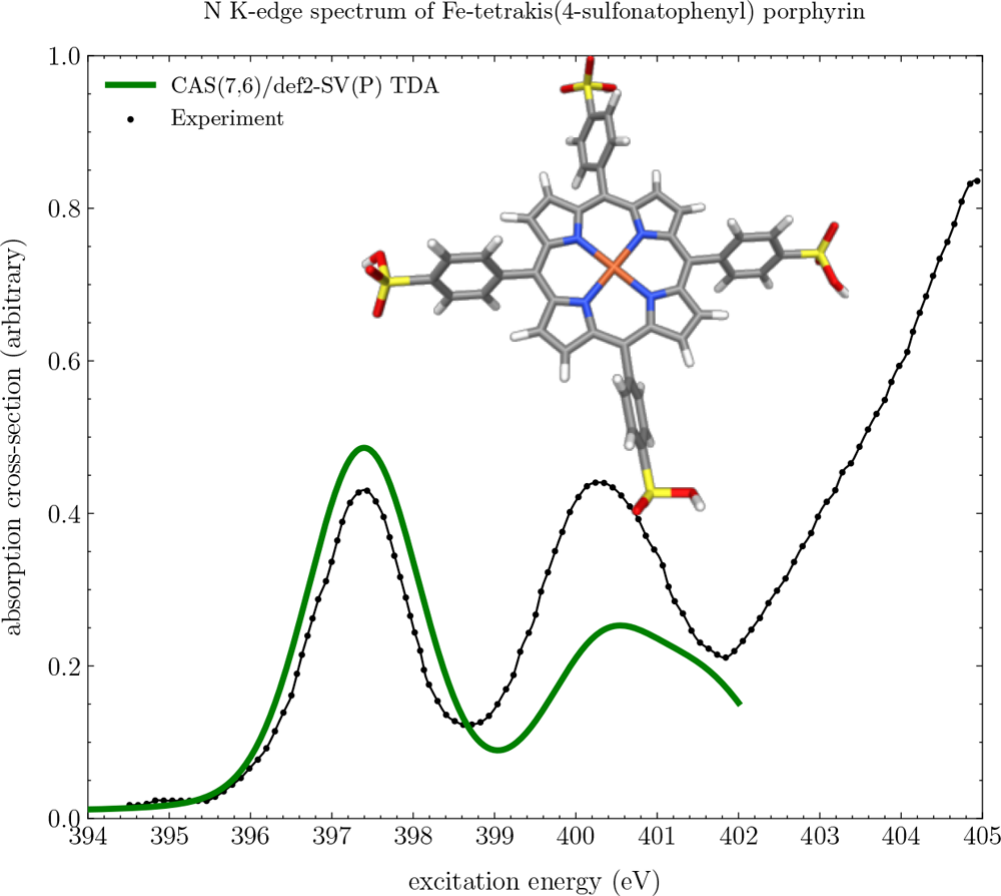
MC-TDA CPP simulated XA spectrum of Fe-tetrakis(4-sulfonatophenyl)porphyrin with a CAS(7,6)/def2-SV(P) wave function. The frequency range is 400–420 eV at a resolution of 200 frequencies. A post-CPP Gaussian broadening with an HWHW of 0.9 eV has been applied. The theoretical spectrum has been red-shifted by 18 eV to align with experiment. The experimental line is from ref (40).

Shown in Figure 10 is the resulting MC-TDA CPP spectrum, calculated between 400 to 420 eV at a resolution of 200 frequencies using a 0.124 eV dampening factor and subsequent HWHM of 0.9 eV Gaussian broadening, obtaining a final Voigt profile spectral shape.

A shift of −18 eV was applied to the calculated spectrum to align with experiment. The experimental spectrum consists of two distinct features, two broad bands centered around 397 and 400.5 eV, respectively. Both of these peaks are clearly reproduced at this level of theory. Energetically higher is an intense band structure, which is outside the calculated spectral window and hence not reproduced. While being a large molecule to run MC calculations on (consisting of 992 (1762) contracted (primitive) Gaussian basis functions), MultiPsi completed this calculation in roughly 8 h running on 4 MPI nodes.

## 4 Conclusion

The CPP scheme was implemented within the MultiPsi package to be applicable to MCSCF wave functions. As tested against relatively large molecules and active spaces, specifically, for the case of a 91-atom Fe-tetrakis(4-sulfonatophenyl)porphyrin derivative with a CAS(15,11)/def2-SV(P) wave function, this novel implementation demonstrates the benefit of the CPP approach, resolving spectral features in the X-ray region with the same computational effort as the eigenvalue approach does for the lowest lying electronically excited states. The code scales well on modern HPC architecture even up to thousands of cores and is also general, allowing any perturbation operator (and thus linear response properties) as well as a variety of MCSCF expansions, providing, for example, the ability to add core orbitals in the active space for X-ray spectra with only limited increase in the number of configurations.

It is our hope that this provides a useful tool for computational chemists and even experimentalists to explore large molecular systems that have so far been beyond the computational limit at the MCSCF level of theory.

## Supporting Information Available

Molecular geometries used and descriptions of relevant active spaces are collected and a short comparison with previous development where gerade and ungerade trial vectors were not used (PDF)

## References

[ref1] Roos B. O.; Taylor P. R.; Sigbahn P. E. A complete active space SCF method (CASSCF) using a density matrix formulated super-CI approach. Chem. Phys. 1980, 48, 157–173. 10.1016/0301-0104(80)80045-0.

[ref2] Andersson K.; Malmqvist P.-Å.; Roos B. O. Second-order perturbation theory with a complete active space self-consistent field reference function. J. Chem. Phys. 1992, 96, 1218–1226. 10.1063/1.462209.

[ref3] Angeli C.; Cimiraglia R.; Evangelisti S.; Leininger T.; Malrieu J.-P. Introduction of n-electron valence states for multireference perturbation theory. J. Chem. Phys. 2001, 114, 10252–10264. 10.1063/1.1361246.

[ref4] Schreiber M.; Silva-Junior M. R.; Sauer S. P. A.; Thiel W. Benchmarks for electronically excited states: CASPT2, CC2, CCSD, and CC3. J. Chem. Phys. 2008, 128, 134110 10.1063/1.2889385. 18397056

[ref5] Persico M.; Granucci G. An overview of nonadiabatic dynamics simulations methods, with focus on the direct approach versus the fitting of potential energy surfaces. Theor. Chem. Acc. 2014, 133, 1526 10.1007/s00214-014-1526-1.

[ref6] Montorsi F.; Segatta F.; Nenov A.; Mukamel S.; Garavelli M. Soft X-ray Spectroscopy Simulations with Multiconfigurational Wave Function Theory: Spectrum Completeness, Sub-eV Accuracy, and Quantitative Reproduction of Line Shapes. J. Chem. Theory Comput. 2022, 18, 1003–1016. 10.1021/acs.jctc.1c00566. 35073066 PMC8830047

[ref7] Lundberg M.; Delcey M. G. In Transition Metals in Coordination Environments: Computational Chemistry and Catalysis Viewpoints; Broclawik E., Borowski T., Radoń M., Eds.; Springer International Publishing: Cham, 2019; pp 185–217.

[ref8] Levine B. G.; Ko C.; Quenneville J.; Martínez T. J. Conical intersections and double excitations in time-dependent density functional theory. Mol. Phys. 2006, 104, 1039–1051. 10.1080/00268970500417762.

[ref9] Jørgensen P.; Jensen H. J. A.; Olsen J. Linear response calculations for large scale multiconfiguration self-consistent field wave functions. J. Chem. Phys. 1988, 89, 3654–3661. 10.1063/1.454885.

[ref10] Helmich-Paris B. Benchmarks for Electronically Excited States with CASSCF Methods. J. Chem. Theory Comput. 2019, 15, 4170–4179. 10.1021/acs.jctc.9b00325. 31136706 PMC6620717

[ref11] Norman P.; Ruud K.; Saue T. Principles and practices of molecular properties: Theory, modeling, and simulations; John Wiley & Sons: 2018.

[ref12] Barth A.; Schirmer J. Theoretical core-level excitation spectra of N2 and CO by a new polarisation propagator method. J. Phys. B: At. Mol. Phys. 1985, 18, 867 10.1088/0022-3700/18/5/008.

[ref13] Herbst M. F.; Fransson T. Quantifying the error of the core-valence separation approximation. J. Chem. Phys. 2020, 153, 054114 10.1063/5.0013538. 32770930

[ref14] Helmich-Paris B. Simulating X-ray absorption spectra with complete active space self-consistent field linear response methods. Int. J. Quantum Chem. 2021, 121, e26559 10.1002/qua.26559.

[ref15] Repisky M.; Konecny L.; Kadek M.; Komorovsky S.; Malkin O. L.; Malkin V. G.; Ruud K. Excitation energies from real-time propagation of the four-component Dirac-Kohn-Sham equation. J. Chem. Theory Comput. 2015, 11, 980–991. 10.1021/ct501078d. 26579752

[ref16] Ekström U.; Norman P.; Carravetta V.; Ågren H. Polarization propagator for X-ray spectra. Phys. Rev. Lett. 2006, 97, 143001 10.1103/PhysRevLett.97.143001. 17155244

[ref17] Jiemchooroj A.; Norman P. Electronic circular dichroism spectra from the complex polarization propagator. J. Chem. Phys. 2007, 126, 134102 10.1063/1.2716660. 17430011

[ref18] Jiemchooroj A.; Ekström U.; Norman P. Near-edge x-ray absorption and natural circular dichroism spectra of L-alanine: A theoretical study based on the complex polarization propagator approach. J. Chem. Phys. 2007, 127, 165104 10.1063/1.2800024. 17979397

[ref19] Pedersen M. N.; Hedegard E. D.; Olsen J. M. H.; Kauczor J.; Norman P.; Kongsted J. Damped response theory in combination with polarizable environments: The polarizable embedding complex polarization propagator method. J. Chem. Theory Comput. 2014, 10, 1164–1171. 10.1021/ct400946k. 26580189

[ref20] Gross E.; Kohn W. Local density-functional theory of frequency-dependent linear response. Phys. Rev. Lett. 1985, 55, 2850 10.1103/PhysRevLett.55.2850. 10032255

[ref21] Gross a. E.; Dobson J.; Petersilka M. Density functional theory of time-dependent phenomena. Density functional theory II 1996, 181, 81–172. 10.1007/BFb0016643. 10061664

[ref22] Maitra N. T.; Zhang F.; Cave R. J.; Burke K. Double excitations within time-dependent density functional theory linear response. J. Chem. Phys. 2004, 120, 5932–5937. 10.1063/1.1651060. 15267474

[ref23] Hettema H.; Jensen H. J. A.; Jørgensen P.; Olsen J. Quadratic response functions for a multiconfigurational self-consistent field wave function. J. Chem. Phys. 1992, 97, 1174–1190. 10.1063/1.463245.

[ref24] Aidas K.; Angeli C.; Bak K. L.; Bakken V.; Bast R.; Boman L.; Christiansen O.; Cimiraglia R.; Coriani S.; Dahle P.; Dalskov E. K.; Ekström U.; Enevoldsen T.; Eriksen J. J.; Ettenhuber P.; Fernández B.; Ferrighi L.; Fliegl H.; Frediani L.; Hald K.; Halkier A.; Hättig C.; Heiberg H.; Helgaker T.; Hennum A. C.; Hettema H.; Hjertenæs E.; Høst S.; Høyvik I.-M.; Iozzi M. F.; Jansík B.; Jensen H. J. Aa.; Jonsson D.; Jørgensen P.; Kauczor J.; Kirpekar S.; Kjærgaard T.; Klopper W.; Knecht S.; Kobayashi R.; Koch H.; Kongsted J.; Krapp A.; Kristensen K.; Ligabue A.; Lutnæs O. B.; Melo J. I.; Mikkelsen K. V.; Myhre R. H.; Neiss C.; Nielsen C. B.; Norman P.; Olsen J.; Olsen J. M. H.; Osted A.; Packer M. J.; Pawlowski F.; Pedersen T. B.; Provasi P. F.; Reine S.; Rinkevicius Z.; Ruden T. A.; Ruud K.; Rybkin V. V.; Sałek P.; Samson C. C. M.; de Merás A. S.; Saue T.; Sauer S. P. A.; Schimmelpfennig B.; Sneskov K.; Steindal A. H.; Sylvester-Hvid K. O.; Taylor P. R.; Teale A. M.; Tellgren E. I.; Tew D. P.; Thorvaldsen A. J.; Thøgersen L.; Vahtras O.; Watson M. A.; Wilson D. J. D.; Ziolkowski M.; Ågren H. The Dalton quantum chemistry program system. WIREs Comput. Mol. Sci. 2014, 4, 269–284. 10.1002/wcms.1172. PMC4171759 25309629

[ref25] Kauczor J.; Jørgensen P.; Norman P. On the efficiency of algorithms for solving Hartree-Fock and Kohn-Sham response equations. J. Chem. Theory Comput. 2011, 7, 1610–1630. 10.1021/ct100729t. 26596429

[ref26] Rinkevicius Z.; Li X.; Vahtras O.; Ahmadzadeh K.; Brand M.; Ringholm M.; List N. H.; Scheurer M.; Scott M.; Dreuw A.; Norman P. VeloxChem: APython-driven density-functional theory program for spectroscopy simulations in high-performance computing environments. WIREs Comput. Mol. Sci. 2020, 10, e1457 10.1002/wcms.1457.

[ref27] Delcey M. G. MultiPsi: A python-driven MCSCF program for photochemistry and spectroscopy simulations on modern HPC environments. WIREs Comput. Mol. Sci. 2023, 10.1002/wcms.1675.

[ref28] Norman P.; Bishop D. M.; Jørgen Aa.; Jensen H.; Oddershede J. Near-resonant absorption in the time-dependent self-consistent field and multiconfigurational self-consistent field approximations. J. Chem. Phys. 2001, 115, 10323–10334. 10.1063/1.1415081.

[ref29] Norman P. A perspective on nonresonant and resonant electronic response theory for time-dependent molecular properties. Phys. Chem. Chem. Phys. 2011, 13, 20519–20535. 10.1039/c1cp21951k. 21970894

[ref30] Werner H.-J.; Knowles P. J. A second order multiconfiguration SCF procedure with optimum convergence. J. Chem. Phys. 1985, 82, 5053–5063. 10.1063/1.448627.

[ref31] Hu C.; Sugino O.; Watanabe K. Performance of Tamm-Dancoff approximation on nonadiabatic couplings by time-dependent density functional theory. J. Chem. Phys. 2014, 140, 054106 10.1063/1.4862904. 24511921

[ref32] Helgaker T. U.; Almlöf J.; Jensen H. J. A.; Jørgensen P. Molecular Hessians for large-scale MCSCF wave functions. J. Chem. Phys. 1986, 84, 6266–6279. 10.1063/1.450771.

[ref33] Helmich-Paris B. CASSCF linear response calculations for large open-shell molecules. J. Chem. Phys. 2019, 150, 174121 10.1063/1.5092613. 31067879

[ref34] Kauczor J.; Norman P. Efficient calculations of molecular linear response properties for spectral regions. J. Chem. Theory Comput. 2014, 10, 2449–2455. 10.1021/ct500114m. 26580765

[ref35] Andersen J. H.; Nanda K. D.; Krylov A. I.; Coriani S. Probing Molecular Chirality of Ground and Electronically Excited States in the UV-vis and X-ray Regimes: An EOM-CCSD Study. J. Chem. Theory Comput. 2022, 18, 1748–1764. 10.1021/acs.jctc.1c00937. 35187935

[ref36] Martin R. L. Natural transition orbitals. J. Chem. Phys. 2003, 118, 4775–4777. 10.1063/1.1558471.

[ref37] Yano J.; Yachandra V. Mn4Ca cluster in photosynthesis: where and how water is oxidized to dioxygen. Chem. Rev. 2014, 114, 4175–4205. 10.1021/cr4004874. 24684576 PMC4002066

[ref38] Delcey M. G.; Lindblad R.; Timm M.; Bülow C.; Zamudio-Bayer V.; von Issendorff B.; Lau J. T.; Lundberg M. Soft X-ray signatures of cationic manganese-oxo systems, including a high-spin manganese(v) complex. Phys. Chem. Chem. Phys. 2022, 24, 3598–3610. 10.1039/D1CP03667J. 35103264

[ref39] Malmqvist P.-Å.; Rendell A.; Roos B. O. The restricted active space self-consistent-field method, implemented with a split graph unitary group approach. J. Phys. Chem. 1990, 94, 5477–5482. 10.1021/j100377a011.

[ref40] Yamashige H.; Matsuo S.; Kurisaki T.; Perera R. C.; Wakita H. Electronic structure analysis of iron (III)-porphyrin complexes by X-ray absorption spectra at the C, N and Fe K-edges. Anal. Sci. 2005, 21, 309–314. 10.2116/analsci.21.309. 15790118

