# Supplementary material for: Complex Linear Response Functions for a Multiconfigurational Self-Consistent Field Wave Function in a High Performance Computing Environment
Source: J Chem Theory Comput. 2023 Aug 19;19(17):5924–37. doi: 10.1021/acs.jctc.3c00317 (PMC10500980; doi:10.1021/acs.jctc.3c00317)
Supplement: Supplementary file 1 — ct3c00317_si_001.pdf [file ct3c00317_si_001.pdf]

Supplementary Information: Complex linear response functions for a multiconfigurational self-consistent field wave function in a high performance computing environment

Mikael Scott[1] and Mickael Delcey[1,2]

[1)] *Division of Theoretical Chemistry and Biology, School of Engineering Sciences in Chemistry, Biotechnology and Health, KTH Royal Institute of Technology, SE-106 91 Stockholm, Sweden*

[2)] *Division of Theoretical Chemistry, Department of Chemistry, Lund University, SE-221 00 Lund, Sweden*

(Dated: 7 August 2023)

S1. CONVERGENCE OF A MC-CPP EQUATION USING G/U AND NON-G/U SUBSPACE VECTORS

The main benefit of the use of gerade/ungerade vectors is faster and more reliable convergence. This is illustrated in Fig. S1 where the g/u solver of MultiPsi is compared to the traditional solver as implemented in the software package Dalton.[1] Here, we note that the traditional solver does not converge to a residual norm of less than 1e-3. The initial residual norm is lower in the non-g/u case because the solver (Dalton v2018.alpha(2018)) starts by solving the two lowest eigenvalues and uses this for the guess. MultiPsi can converge to an almost arbitrarily high precision (tested to 1e-9 residual) while non-g/u convergence often hits a limit significantly above 1e-3, after which the residual norm does not decrease regardless of number of trial vectors.

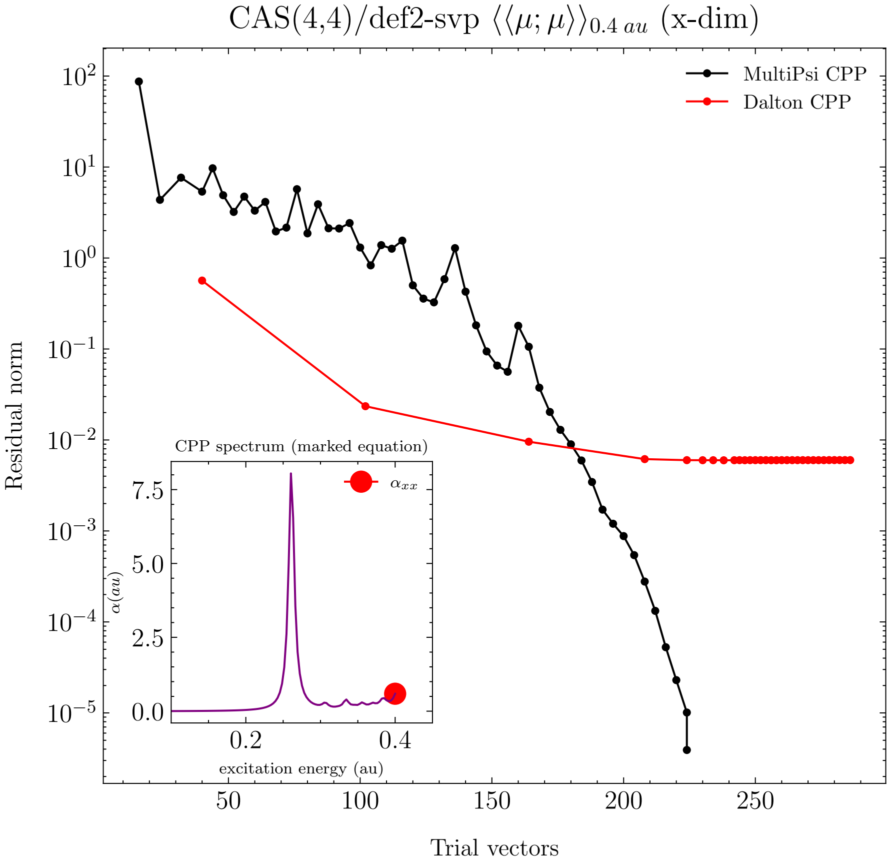

FIG. S1. Illustrative example of how g/u improves convergence compared to non-g/u methods. Test-case is the molecule $(CH_2{=}CH_2)_2N\text{-}CH_2\text{-}CH_2\text{-}CH_3$, using a CAS(4,4)/def2-SVP wave function at an excitation energy of 0.4 a.u. (marked in red in the inset).

All geometries are reported in Ångström.

S2. PORHYRIN-A: GEOMETRY

6 −4

N	0.29280	1.69514	−0.14098
N	−1.96480	0.17871	−0.39409
N	1.91405	−0.77441	0.37863
N	−0.33521	−2.20932	0.10749
C	−0.62213	2.77421	−0.25062
C	−2.58026	1.37836	−0.53143
C	1.59160	2.28448	0.00384
C	−2.97318	−0.74522	−0.48233
C	−1.93510	2.68019	−0.44337
C	0.08245	4.04166	−0.11495
C	−3.93817	1.22713	−0.72314
C	1.39903	3.75670	0.06891
C	−4.18831	−0.10912	−0.69811
C	2.77659	1.66754	0.25911
C	−2.86722	−2.09408	−0.34817
C	2.91067	0.16599	0.46473
C	−1.59389	−2.80663	−0.06386
C	2.52567	−1.98572	0.52900
C	0.52311	−3.29936	0.28946
C	1.94851	−3.18753	0.47898
C	4.12549	−0.51957	0.64768
C	−1.47108	−4.13748	0.02029
C	3.87381	−1.83808	0.70918
C	−0.15206	−4.44174	0.24443
H	−0.33601	5.04003	−0.11860
H	−4.67893	2.00594	−0.85139
H	2.12984	4.53251	0.24563
H	−5.16852	−0.55591	−0.80162
H	5.14483	−0.18429	0.69920
H	−2.25229	−4.87592	−0.09103
H	4.62465	−2.60811	0.83635
H	0.25749	−5.43848	0.34583
C	3.93864	2.34317	0.30471
C	4.99410	2.14590	1.11553
C	6.29260	2.84522	1.01285
C	6.46596	3.78304	0.08523
C	5.33768	4.11549	−0.79574
C	4.17651	3.44231	−0.68705
C	−2.75654	3.91713	−0.53167
C	−3.09690	4.62093	0.63454
C	−3.84559	5.79862	0.55275
C	−4.25023	6.28853	−0.69409
C	−3.91223	5.58924	−1.85985
C	−3.17297	4.40415	−1.77950
C	−4.07618	−2.96778	−0.47442
C	−4.76852	−3.11077	−1.78511
C	−5.78841	−3.97706	−1.91903
C	−6.24562	−4.77771	−0.75455
C	−4.50193	−3.69689	0.58035
C	−5.64049	−4.63144	0.43658
C	2.76639	−4.42424	0.56669
C	2.95736	−5.08222	1.78878
C	3.76000	−6.22707	1.84635
C	3.37684	−4.91958	−0.59481
C	4.17240	−6.06423	−0.53664
C	4.36871	−6.72020	0.68333
H	−4.00295	−3.61996	1.54234
H	−4.42865	−2.54095	−2.64461
H	−6.27219	−4.09724	−2.88307
H	−5.96262	−5.21267	1.29505
H	2.49685	−4.70195	2.69201
H	3.91121	−6.72446	2.79529

H	3.23705	−4.41505	−1.54355
H	4.89352	1.49672	1.98052
H	7.09750	2.58025	1.69184
H	3.41925	3.69901	−1.42067
H	5.45559	4.87518	−1.56207
H	−2.78323	4.25542	1.60464
H	−4.10382	6.33228	1.45872
H	−4.21511	5.96552	−2.82859
H	−2.90845	3.87700	−2.68764
S	−7.58373	−5.95777	−0.94218
S	−5.18457	7.81536	−0.79263
H	4.63964	−6.43527	−1.44009
S	5.41002	−8.17745	0.74667
S	8.05562	4.59017	−0.10355
O	5.14137	−9.11684	−0.43861
O	5.06083	−9.08658	1.93432
O	−7.12272	−7.38717	−0.61870
O	−8.69810	−5.74614	0.09305
O	−4.29416	9.00797	−1.17193
O	−6.19699	7.80018	−1.94767
O	8.59572	5.14709	1.22163
O	9.14633	3.57679	−0.48351
O	7.11456	−7.98393	0.80011
O	−8.38140	−6.07875	−2.45693
O	−6.07759	8.33078	0.57980
O	8.21789	5.86208	−1.24381
Fe	0.01496	−0.27104	−0.10995
N	−0.10098	−0.73762	−2.02739
O	0.72795	−0.33933	−2.78651

S3. FE-TETRAKIS(4-SULFONATOPHENYL) PORPHYRIN: GEOMETRY

Geometry optimzed at the B3LYP/def2-sv(p) level of theory.

1 6

N	0.48610245711134	1.79229104449628	−0.08611506824921
N	−1.96600944798322	0.18331059569142	−0.30157482491107
N	2.08015877416946	−0.66158209511126	0.19865652794426
N	−0.38454544968358	−2.26979854250710	0.06395963272223
C	−0.42016526628912	2.81459910012627	−0.19961416733347
C	−2.53865547123214	1.43002993340837	−0.32411663544381
C	1.73486361789613	2.36033670702835	−0.04831238634803
C	−2.98753735733488	−0.73012692391591	−0.34393279272841
C	−1.83380462542242	2.66568380168607	−0.29122445714689
C	0.27932628256895	4.07848010770861	−0.24306041430177
C	−3.97775235695081	1.30033897211719	−0.36065012781781
C	1.61430400849980	3.79738948511652	−0.13765682707795
C	−4.25558646122206	−0.03993277527769	−0.38107435002312
C	2.96387107436845	1.65820829139943	0.09055856746081
C	−2.83796858258434	−2.14498233578598	−0.31695790907094
C	3.10548142997469	0.24764875687376	0.22670893439355
C	−1.61313252619072	−2.84560281837308	−0.14110208047910
C	2.64150500129002	−1.90723332217080	0.32940674009809
C	0.52658780204464	−3.28892949272526	0.15953079100735
C	1.93311286912572	−3.13817554986934	0.31813575857161
C	4.36413137245714	−0.44642318724543	0.38087148561159
C	−1.47192251430258	−4.28282841778508	−0.18902918036083
C	4.07405787120402	−1.78094446763580	0.46570800551490
C	−0.14593370573931	−4.55798199488067	0.00413151095419
H	−0.17850945775542	5.05929157171914	−0.34910117525992
H	−4.69268401120608	2.12012900457494	−0.35197377272900
H	2.43652881778310	4.50928531140888	−0.11886777401320
H	−5.23665083154929	−0.51042726015827	−0.39213505936686
H	5.34836122409962	0.01477712819712	0.41759881146813
H	−2.27103997075661	−4.99744699833640	−0.37124516322780
H	4.77660221295136	−2.59988372726597	0.60445338815596
H	0.32885853912024	−5.53644799960307	0.01042473755256
C	4.21768390291497	2.47198396728109	0.07291552581630
C	5.04007243962532	2.56025569947249	1.21303209091260
C	6.21524463840907	3.31294951781587	1.18309650201493
C	6.57069030405091	3.97159797347628	0.00276103412326
C	5.77933911073276	3.89092954880138	−1.14500876288044
C	4.59775061789071	3.14806217315250	−1.10219528793407
C	−2.64441676890677	3.91778684375114	−0.36689892047511
C	−2.65893055262583	4.82547071176213	0.71033778482610
C	−3.41184214297971	5.99863179199626	0.64065233878699
C	−4.14197901365683	6.26936784934031	−0.51951054524869
C	−4.13166354641370	5.39475766122489	−1.60901893426291
C	−3.39147406706334	4.21409358203989	−1.52348735830636
C	−4.06325147048315	−2.97547412084658	−0.51722100639006
C	−4.73734886644454	−2.94318752850108	−1.75278677735656
C	−5.83069474197768	−3.77992521270540	−1.98853324891798
C	−6.25891836970558	−4.63718924264062	−0.97293796946848
C	−4.53261295607868	−3.83131203566620	0.49905251520333
C	−5.63752243534427	−4.65477121847652	0.27916545072334
C	2.72552531735130	−4.39854288351192	0.44670850484561
C	2.57607741237736	−5.21517842283829	1.58410854119790
C	3.28564974762402	−6.41161824930547	1.69764484594025
C	3.60480122561887	−4.79823393122678	−0.57698842449365
C	4.30017872573438	−6.00555536272288	−0.48234234857448
C	4.12568845895645	−6.80272440280398	0.65131362354698
H	−4.03484525423120	−3.84455280002478	1.47292660092506
H	−4.38409561680632	−2.28119246674146	−2.54852257993728
H	−6.34875252127420	−3.77434440673586	−2.95066725946002
H	−6.01723561510634	−5.30589204623705	1.07081416746822
H	1.91001489123621	−4.90387272543386	2.39399254317645
H	3.19478579822633	−7.03677046233919	2.58948468727359
H	3.72898821194131	−4.17191441199179	−1.46504941829427
H	4.75376950308595	2.04460200288766	2.13409611510988

H	6.85956322501216	3.37959712827652	2.06354713945030
H	3.97646656726969	3.07269951267949	−1.99920592223171
H	6.09575489759890	4.39494154970675	−2.06152303783440
H	−2.08551500014533	4.60635657923455	1.61546699338653
H	−3.42357137796676	6.70739058085159	1.47267184478707
H	−4.68735699886643	5.64599837752928	−2.51586137011454
H	−3.37108477969066	3.52846631579367	−2.37536240950206
S	−7.63679228633992	−5.74513113353955	−1.28798999181418
S	−5.09517069812157	7.78796700617332	−0.62500399260830
H	4.98007581799241	−6.32632244444465	−1.27566157420531
S	5.01100090886061	−8.35999595533817	0.77252858339584
S	8.09496556861984	4.91875469475042	−0.05449888554939
O	3.87212924738251	−9.39685561293969	0.20125417438617
O	5.19464062937402	−8.65520976378076	2.18093069559995
O	−8.31844897730178	−6.04372608532479	−0.03232411790063
O	−8.34100094656110	−5.26608463522259	−2.46389459799454
O	−4.56259938234005	8.75064502682021	0.33501003007057
O	−5.28270118517540	8.09472274235357	−2.03084722400705
O	7.54228981128580	6.35253074265172	0.52769093299845
O	9.02742063770668	4.38801236353833	0.93582485711479
O	6.10344676888663	−8.35444920833402	−0.19527588559045
O	−6.78582895685232	−7.09097681451193	−1.70024928785219
O	−6.53357261325613	7.26286987250022	−0.03125419855752
O	8.45322225848420	5.10066749812174	−1.44967716831393
Fe	0.05237497881934	−0.23833942566481	−0.01219624466787
H	4.21153397266029	−9.80898735065992	−0.61935137049069
H	8.13383114296635	6.63890953184607	1.25416112339309
H	−7.16490556745789	−7.85260847368250	−1.21416689494808
H	−6.76639344798403	7.80981458545873	0.74700614414373

S4. FE-TETRAKIS(4-SULFONATOPHENYL) PORPHYRIN: ACTIVE SPACE CAS(7,6)

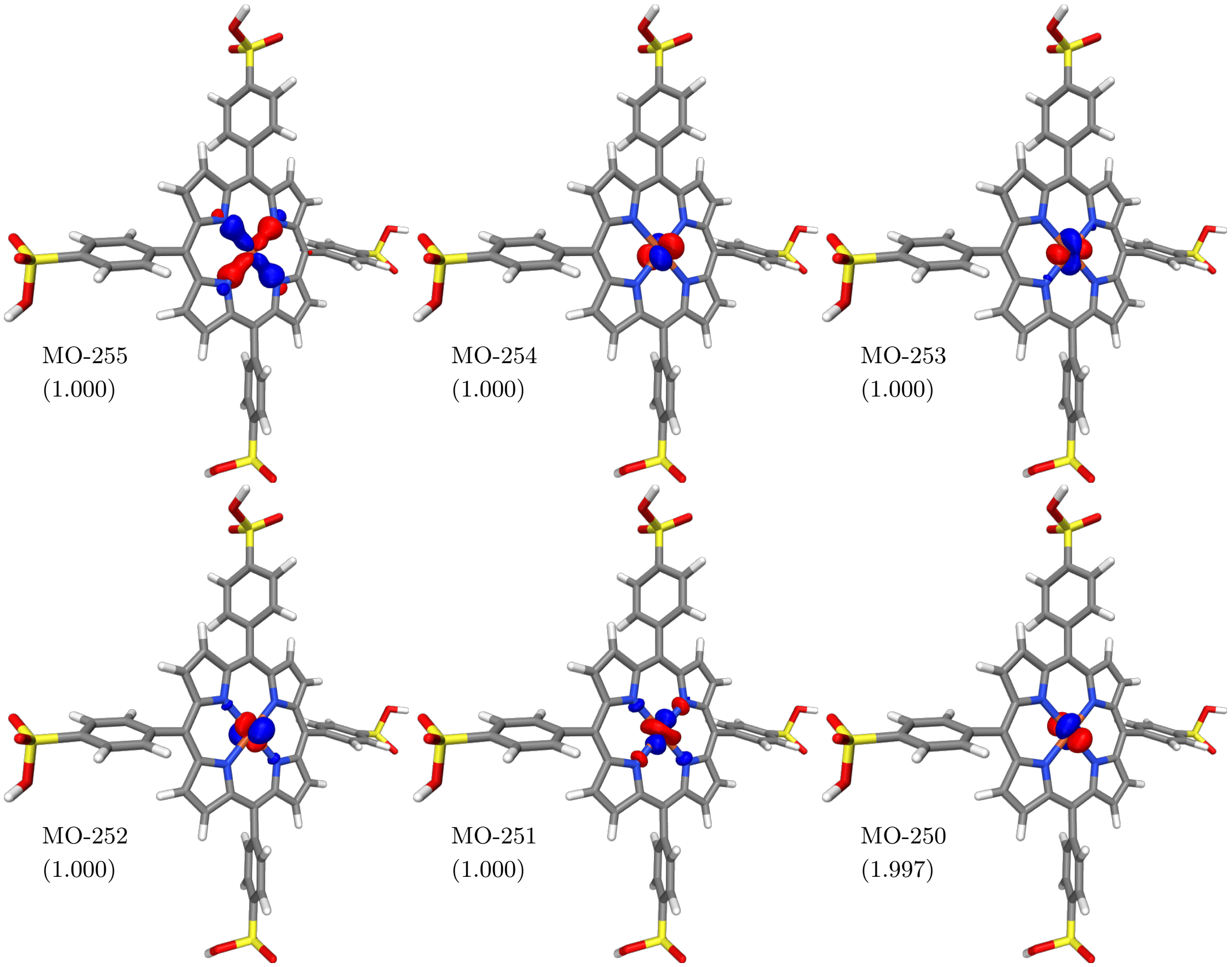

FIG. S2. Fe-tetrakis(4-sulfonatophenyl) porphyrin CAS(7,6) active space. Occupation numbers in parenthesis.

S5. BENZENE: GEOMETRY

0 1			
C	−4.748356	−0.043254	−9.161550
C	−5.907241	−0.398590	−8.461619
C	−3.589470	0.312083	−8.461619
H	−6.813850	−0.677085	−9.009129
H	−2.682862	0.590578	−9.009129
C	−5.907241	−0.398590	−7.061905
C	−3.589470	0.312082	−7.061905
H	−6.813850	−0.677085	−6.514395
H	−2.682862	0.590578	−6.514395
C	−4.748356	−0.043254	−6.361975
H	−4.748356	−0.043254	−5.266867
H	−4.748356	−0.043254	−10.256657

S6. MNO$^+$ AND MNO$_2^+$: GEOMETRY

```
5 1
Mn 0.0 0.0 0.0
O 0.0 0.0 1.675
```

```
3 1
Mn    0.000000    0.051620    0.000000
O     0.000000    0.848380    1.369435
O     0.000000    0.848380   −1.369435
```

S7. MNO$^+$ AND MNO$_2^+$: ACTIVE SPACE

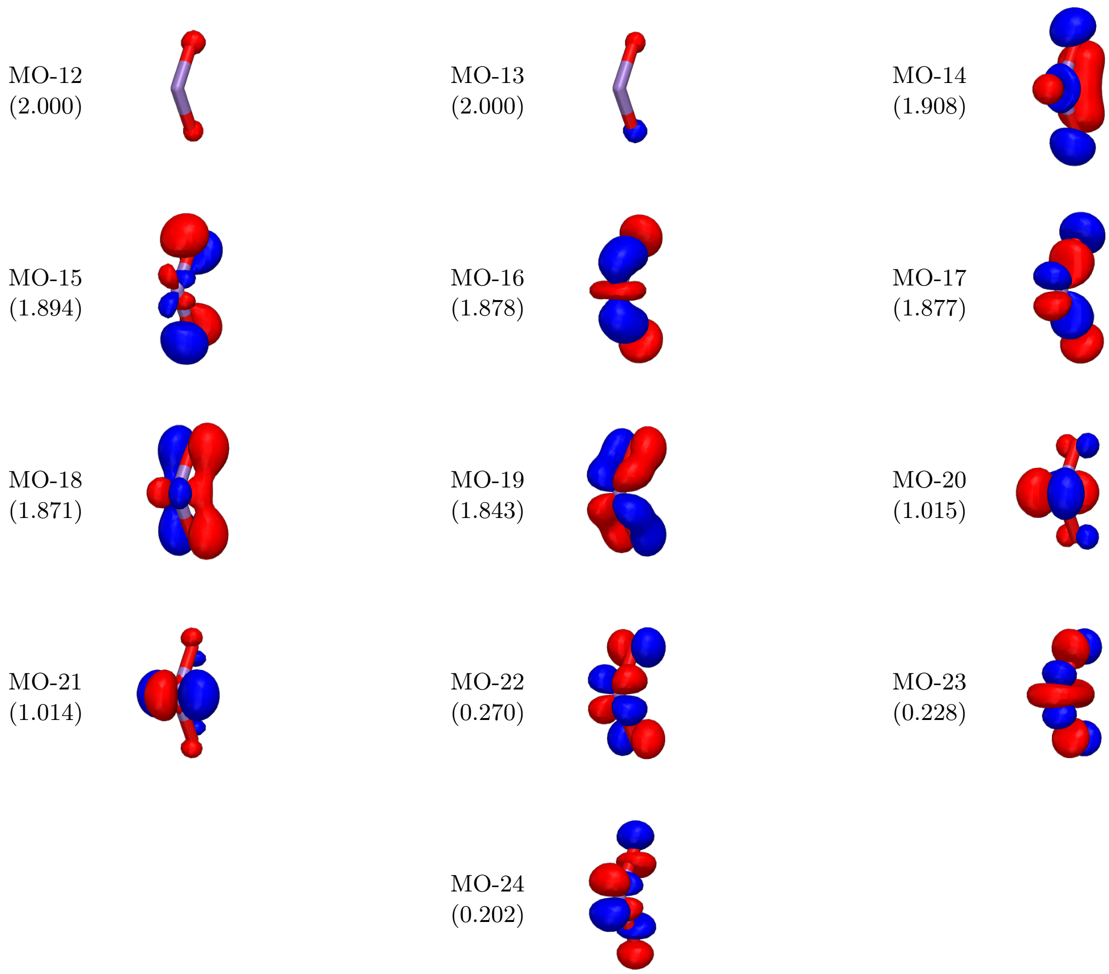

FIG. S3. MnO_2^+ active spaces, without O 1s orbitals: CAS(14,11), with O 1s orbitals: CAS(18,13). Occupation numbers in parenthesis.

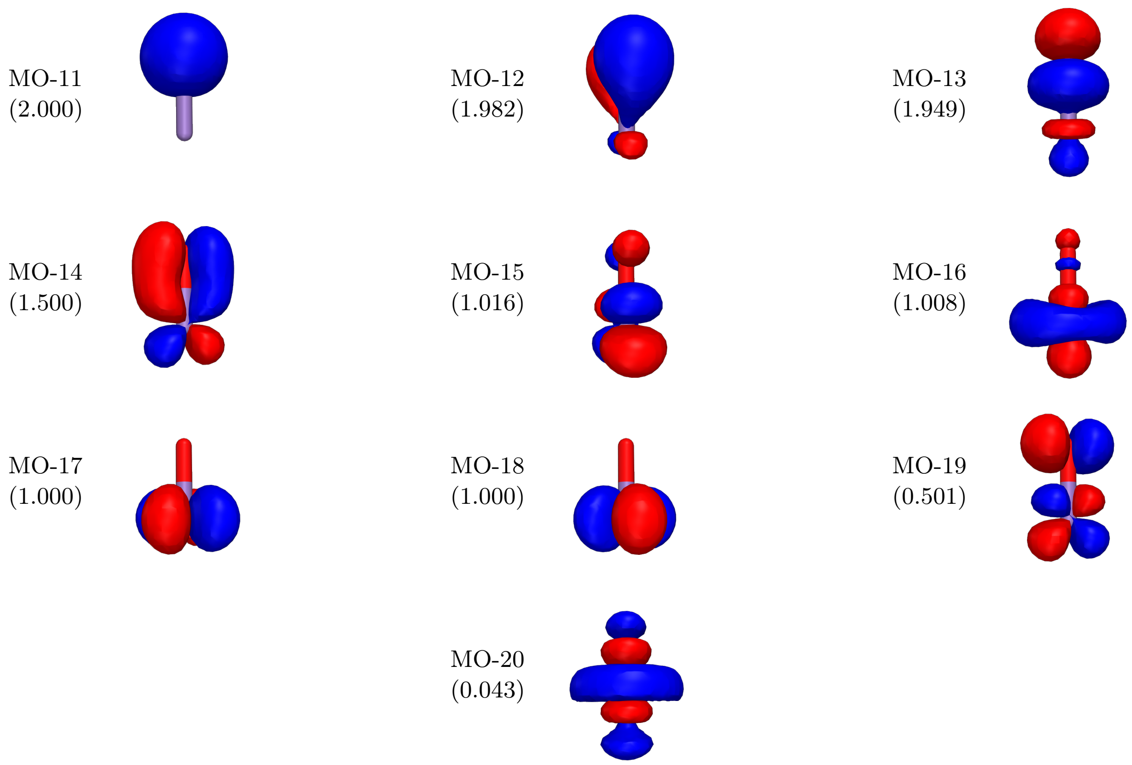

FIG. S4. MnO^+ active spaces, without O 1s orbitals: CAS(10,9), with O 1s orbitals: CAS(12,10). Occupation numbers in parenthesis.

REFERENCES

[1]K. Aidas, C. Angeli, K. L. Bak, V. Bakken, R. Bast, L. Boman, O. Christiansen, R. Cimiraglia, S. Coriani, P. Dahle, E. K. Dalskov, U. Ekström, T. Enevoldsen, J. J. Eriksen, P. Ettenhuber, B. Fernández, L. Ferrighi, H. Fliegl, L. Frediani, K. Hald, A. Halkier, C. Hättig, H. Heiberg, T. Helgaker, A. C. Hennum, H. Hettema, E. Hjertenæs, S. Høst, I.-M. Høyvik, M. F. Iozzi, B. Jansík, H. J. Aa. Jensen, D. Jonsson, P. Jørgensen, J. Kauczor, S. Kirpekar, T. Kjærgaard, W. Klopper, S. Knecht, R. Kobayashi, H. Koch, J. Kongsted, A. Krapp, K. Kristensen, A. Ligabue, O. B. Lutnæs, J. I. Melo, K. V. Mikkelsen, R. H. Myhre, C. Neiss, C. B. Nielsen, P. Norman, J. Olsen, J. M. H. Olsen, A. Osted, M. J. Packer, F. Pawlowski, T. B. Pedersen, P. F. Provasi, S. Reine, Z. Rinkevicius, T. A. Ruden, K. Ruud, V. V. Rybkin, P. Sałek, C. C. M. Samson, A. S. de Merás, T. Saue, S. P. A. Sauer, B. Schimmelpfennig, K. Sneskov, A. H. Steindal, K. O. Sylvester-Hvid, P. R. Taylor, A. M. Teale, E. I. Tellgren, D. P. Tew, A. J. Thorvaldsen, L. Thøgersen, O. Vahtras, M. A. Watson, D. J. D. Wilson, M. Ziolkowski, and H. Ågren, "v2018.alpha(2018), The Dalton quantum chemistry program system," WIREs Comput. Mol. Sci. **4**, 269–284 (2014).
